# Microfluidic affinity selection of active SARS-CoV-2 virus particles

**DOI:** 10.1126/sciadv.abn9665

**Published:** 2022-09-28

**Authors:** Sachindra S. T. Gamage, Thilanga N. Pahattuge, Harshani Wijerathne, Katie Childers, Swarnagowri Vaidyanathan, Uditha S. Athapattu, Lulu Zhang, Zheng Zhao, Mateusz L. Hupert, Rolf M. Muller, Judy Muller-Cohn, Janet Dickerson, Dylan Dufek, Brian V. Geisbrecht, Harsh Pathak, Ziyan Pessetto, Gregory N. Gan, Junseo Choi, Sunggook Park, Andrew K. Godwin, Malgorzata A. Witek, Steven A. Soper

**Affiliations:** ^1^ Department of Chemistry, The University of Kansas, Lawrence, KS 66045, USA.; ^2^ Center of BioModular Multiscale Systems for Precision Medicine, The University of Kansas, Lawrence, KS 66045, USA.; ^3^ Bioengineering Program, The University of Kansas, Lawrence, KS 66045, USA.; ^4^ BioFluidica Inc., San Diego, CA 92121, USA.; ^5^ Department of Biochemistry and Molecular Biophysics, Kansas State University, Manhattan, KS 66506, USA.; ^6^ Department of Pathology and Laboratory Medicine, University of Kansas Medical Center, Kansas City, KS 66160, USA.; ^7^ Sinochips Diagnostics, Olathe, KS 66061, USA.; ^8^ Department of Radiation Oncology, University of Kansas Medical Center, Kansas City, KS 66160, USA.; ^9^ University of Kansas Cancer Center, University of Kansas Medical Center, Kansas City, KS 66160, USA.; ^10^ Department of Industrial and Mechanical Engineering, Louisiana State University, Baton Rouge, LA 70803, USA.; ^11^ Department of Mechanical Engineering, The University of Kansas, Lawrence, KS 66045, USA.

## Abstract

We report a microfluidic assay to select active severe acute respiratory syndrome coronavirus 2 (SARS-CoV-2) viral particles (VPs), which were defined as intact particles with an accessible angiotensin-converting enzyme 2 receptor binding domain (RBD) on the spike (S) protein, from clinical samples. Affinity selection of SARS-CoV-2 particles was carried out using injection molded microfluidic chips, which allow for high-scale production to accommodate large-scale screening. The microfluidic contained a surface-bound aptamer directed against the virus’s S protein RBD to affinity select SARS-CoV-2 VPs. Following selection (~94% recovery), the VPs were released from the chip’s surface using a blue light light-emitting diode (89% efficiency). Selected SARS-CoV-2 VP enumeration was carried out using reverse transcription quantitative polymerase chain reaction. The VP selection assay successfully identified healthy donors (clinical specificity = 100%) and 19 of 20 patients with coronavirus disease 2019 (COVID-19) (95% sensitivity). In 15 patients with COVID-19, the presence of active SARS-CoV-2 VPs was found. The chip can be reprogrammed for any VP or exosomes by simply changing the affinity agent.

## Teaser

The reported technology selects active SARS-CoV-2 from saliva samples for identification of infectious patients with COVID-19.

## INTRODUCTION

The severe acute respiratory syndrome coronavirus 2 (SARS-CoV-2) causing coronavirus disease 2019 (COVID-19) has been found to be highly infectious resulting in the need for community-based quarantines that were implemented in 2020 and 2021. Individuals considered to be spreaders of active viral particles (VPs) must have an intact viral envelope with accessible receptor binding domains (RBDs) of the spike (S) proteins that can bind to an angiotensin-converting enzyme 2 (ACE2) receptor to mediate cell entry of virions and their replication (*1*). The identification of individuals with active disease is necessary to make informed decisions on whom should or should not be quarantined and for the appropriate length of time to reduce negative socioeconomic consequences (*2*).

**Fig. 1.**
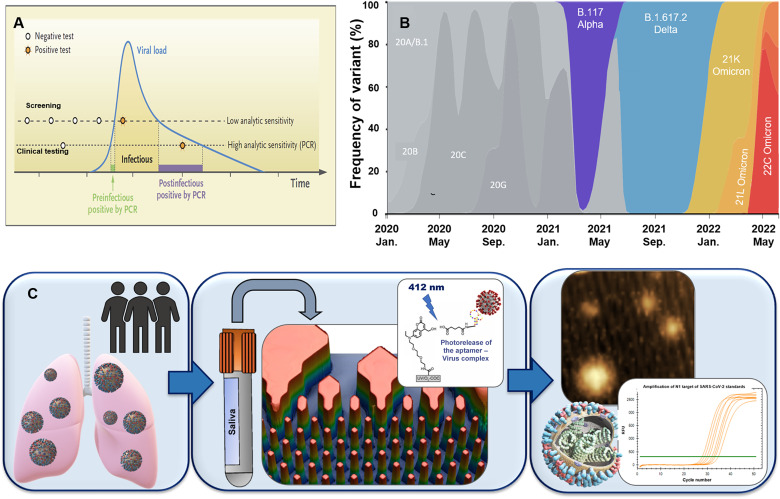
Viral load profile and variant frequency in Kansas and Missouri. (**A**) Hypothetical viral load as a function of disease progression and different testing strategies. From (*2*) *New England Journal of Medicine*, M. J. Mina, R. Parker, D. B. Larremore, Rethinking Covid-19 test sensitivity—a strategy for containment, vol. 383, pg. e120. Copyright © (2020) Massachusetts Medical Society. Reprinted with permission from the Massachusetts Medical Society. (**B**) Frequency of appearance of different SARS-CoV-2 variants identified in Kansas and northeast Missouri between November 2020 and May 2022. Variants are presented as clade and normalized to 100% at each time point. Data are found at www.gisaid.org/phylodynamics/global/nextstrain/. (**C**) Schematic showing the workflow of the reported assay. Saliva samples [COVID-19(+) or COVID-19(−)] are flowed through a microfluidic chip containing an aptamer (affinity agent) that is attached to the surface of the chip through a heterobifunctional linker that can be cleaved using blue light. Following release of intact VPs, the selected particles can be characterized via atomic force microscopy (AFM), transmission electron microscopy (TEM), or nanoparticle tracking analysis (NTA) and subjected to RT-qPCR. In this case, the microfluidic chip selects active VPs that have an accessible ACE2 research binding domain in the S protein.

To mitigate infectious disease spread and enable communal surveillance, different testing platforms have been recognized as important tools for planning proper containment strategies. Large-scale screening allows for early detection of the infection so that prompt quarantine procedures can be implemented (Fig. 1A) (*2*). The challenge is, however, that no test currently available can identify “super spreaders” with active disease who have intact VPs with an accessible ACE2 RBD within the virus’ S protein. Even with widespread vaccinations underway in the United States and globally, testing remains important because of breakthrough cases with the U.S. Centers for Disease Control and Prevention still requiring 10 to 14 days of quarantine for those with positive COVID-19 results irrespective of vaccination status (*3*).

Determining the presence of genomic RNA (gRNA) via polymerase chain reaction (PCR)–based tests from the infiltrating virus has been viewed as an important tool for the control of infectious diseases including COVID-19 and can take on different formats (screening versus clinical) with each having different analytical figures of merit requirements (see Fig. 1A) (*2*). The Food and Drug Administration (FDA) has approved six PCR-based tests for COVID-19 through their emergency use authorization (EUA) mechanism (*4*–*7*). While reverse transcription quantitative PCR (RT-qPCR) is sensitive and specific, it detects the presence of gRNA regardless of its source (i.e., intact VPs that can have either an accessible or nonaccessible RBD on the S protein or free gRNA shed from VPs) (*8*).

Antibody- or antigen-based lateral flow assays have received FDA approval through an EUA as well (*8*, *9*). However, these tests have inferior limits of detection compared to RT-qPCR and high false-negative results (*10*). Tests based on the presence of target antibodies do not provide information about active disease but inform only on whether infection occurred. Antigen tests detect specific protein fragments residing on the surface of the virus, but these tests require high viral loads to minimize false-negative results.

The temporal dynamics of viral load for SARS-CoV-2 has determined that COVID-19 infectivity starts ~12 days (mean) before symptom onset, peaking ~2 days before and 1 day after their onset, but declines rapidly ~7 days after initial symptoms (Fig. 1A) (*11*). Hence, patients following a positive SARS-CoV-2 test may remain in quarantine even if they may not be infectious because PCR, serological, or antigen tests cannot distinguish between active and nonactive disease.

Evolving infectious disease-based tests using microfluidics for the enrichment and selection of VPs and subsequent detection can serve as an enabling platform technology for point-of-care testing (POCT), which would enable more frequent testing to aid in the surveillance of disease spreading. Recently, published reviews have summarized microfluidics’ importance for the analysis of VPs, including SARS-CoV-2 (*12*, *13*). The vast majority of research using microfluidics for VP isolation uses affinity selection, filtration, or dielectrophoresis (*14*).

In terms of detection, Seo *et al.* (*15*) demonstrated the use of graphene as a field-effect transistor decorated with anti-SARS antibodies. The anti–SARS-CoV-2 S antibody used in this study showed no cross-reactivity with Middle East respiratory syndrome–CoV S1 proteins with a detectable signal for ≥100 pg/ml of free S protein, However, this test would not be able to distinguish active from nonactive disease. Methodologies have also reported the detection of COVID-19 based on plasmonic nanoparticles (*16*, *17*). These tests show high sensitivity (>96.6%) and specificity (100%), with a limit of detection of 10 gRNA copies/μl. However, they rely on gRNA signatures without the ability to identify intact VPs. To identify patients with active disease, the approach must detect SARS-CoV-2 VPs that are intact and have an accessible ACE2 RBD in the S protein (Fig. 1A) (*2*).

Affinity selection of biologics offer attractive operational characteristics including the ability to select targets with high purity from a variety of biological samples. Monoclonal antibodies are commonly used for affinity selection. However, in cases of rapidly evolving pandemics, fast development of antibodies specific to a particular virus may be difficult as the time needed for their selection may take ≥6 months. (*18*). Development of antibodies is further complicated by the occurrence of new variants of concern (VOCs) that result from mutations in the gRNA and can result in structural modifications of the RBD of the S protein. As of spring 2022, there were five major VOCs of SARS-CoV-2 globally with different mutations in the RBD: the Alpha variant (i.e., UK variant/B.1.1.7), the Beta variant (i.e., South Africa variant/B.1.351), the Gamma variant (i.e., Brazil variant/P.1), the Delta variant (i.e., India variant/B.1.617.2), and the Omicron variant (B.1.1.529). Omicron had >30 mutations located in the S protein and nearly 15 of those occurring in the RBD (*19*). Figure 1B shows the time appearance of VOCs in KS and MO between November 2020 and May 2022.

Aptamers based on single-stranded nucleic acids (DNA, RNA, or 2′-modified RNA) have several characteristics that make them attractive as affinity selection agents for various applications (*20*–*22*), including quick evolvability using in vitro selection. If appropriately selected, then aptamers can exhibit high affinity and specificity to their cognizant targets with favorable binding affinities (*22*, *23*). Their small size can allow for accessing the epitope’s cavity, which may contain mutated residues that allow for discrimination of subtle molecule differences. Because the start of the COVID-19 pandemic, reports (*24*–*27*) have appeared discussing the development of anti–SARS-CoV-2 aptamers targeting the RBD of the S protein. For example, a DNA aptamer targeting the S protein RBD [dissociation rate constant (*K*_d_) = 5.8 nM] in the SARS-CoV-2 viral envelope has been reported (*26*), which was used in these studies.

Peinetti *et al.* (*24*) developed an assay using a DNA aptamer to select SARS-CoV-2 and incorporated it into a nanopore for VP detection and showed a limit of detection of 1 × 10^4^ particles/ml. However, when using saliva samples, it required 100× dilution and only 15 μl of saliva could be processed, which can generate high false-negative rates for detection of low viral loads.

We describe a COVID-19 technology appended to RT-qPCR to allow for selective sourcing of gRNA to discriminate between individuals with active and nonactive disease (see Fig. 1C for schematic of the workflow). The technology is highly innovative and uses a microfluidic to (i) affinity select SARS-CoV-2 VPs directly from a clinical sample (i.e., saliva) using a surface-bound aptamer directed against the RBD of the SARS-CoV-2 S protein, which was surface-immobilized to a plastic chip containing 1.5 million micropillars (VP selection chip); (ii) photo release of the selected VPs using a blue light-emitting diode (LED) (*28*); and (iii) gRNA isolation from intact and affinity-selected VPs followed by RT-qPCR for quantification.

Because of the affinity selection of intact VPs with an accessible RBD of the S protein, the technology when coupled to RT-qPCR provided information about patients with active versus nonactive disease that could provide more concise information about determining the length of quarantines for those with positive RT-qPCR results. The VP selection chips were fabricated in thermoplastics via injection molding, which provides for high-scale production at low cost allowing for rapid dissemination into testing environments, even for POCT (*29*).

## RESULTS

### VP selection chip and blue light release

**Fig. 2.**
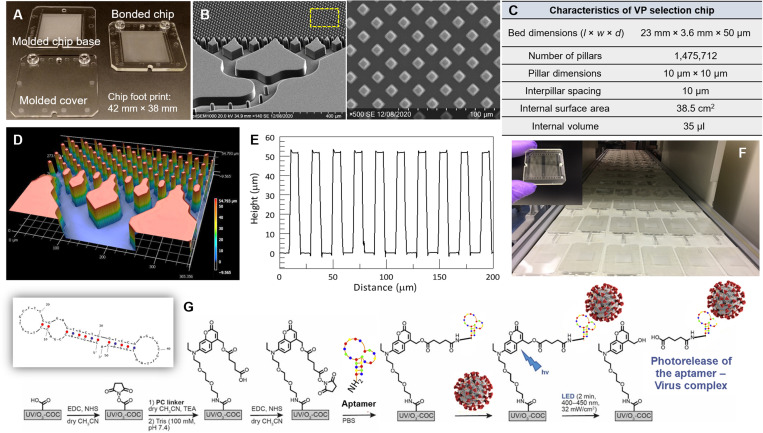
The VP selection chip and covalent attachment of the affinity agent. (**A**) Micrographs of the VP selection chip, cover plate, and assembled VP selection chip. (**B**) Scanning electron microscopies (SEMs) of several selection beds in the VP selection chip. Shown is the fluidic input/output feed network into several beds and a high-resolution SEM of one bed with its micropillars. (**C**) Summary of the operational characteristics of the VP selection chip. (**D**) Rapid scanning confocal image of a section of the VP selection chip. (**E**) Topographical profile of the micropillars and interpillar spacing in the VP selection chip shown in (D). (**F**) Production line of the injection molded VP selection chips. (**G**) Scheme demonstrating covalent attachment of the aptamer via the PC linker to the UV/O_3_-activated COP surface of the plastic chip. Also shown is the secondary structure of the 51–nucleotide (nt) SARS-CoV-2 aptamer (see electrospray ionization for detailed description of this secondary structure).

The VP selection chip contained seven beds connected in parallel with perpendicular inlet and outlet channels arranged in a z-configuration. The chip had ~1.5 million diamond-shaped pillars (10 μm by 10 μm, 10-μm spacing) providing a 38.6-cm^2^ surface area (Fig. 2, A to E). The advantage of diamond-shaped pillars over circular is that diamond structures allow for uniform distance between pillars in every direction, which is important because it maintains a high recovery, which is diffusion-limited. In addition, the packing density of diamond-shaped pillars is higher than circular pillars. The high number of pillars in seven beds (i.e., high surface area) along with the small interpillar spacing (i.e., reduced diffusional distances) allowed for high recovery of VPs and a high dynamic range, respectively, but with a small form factor. Incorporation of seven isolation beds also allowed for high-throughput analysis keeping sample processing time short. On the basis of the available surface area, density of surface-immobilized aptamers, and an average VP size of 150 nm, the theoretical particle load on a chip is 2.2 × 10^11^. The chip was made from cyclic olefin polymer (COP) via injection molding to allow for high-scale production (Fig. 2F) (*29*).

Previously, this chip design was used for the antibody-based selection of extracellular vesicles (*30*). Monte Carlo simulations were used to not only drive the design but also model the performance (*30*). Considering similarities between extracellular vesicles (EVs) and VPs in terms of their size and the display of markers along the surface the nanoparticles, we surmised that our Monte Carlo simulations for EVs could be used to simulate VP transport through the VP selection device. With the pillar spacing used here and the length of the selection beds, we could operate at a volumetric flow rate of 20 μl/min and secure a recovery of >80%, meaning that an input of 200 μl could be processed in 10 min. We should note that while antibodies are attached to the pillar surface randomly, aptamers as used here have a single anchoring point (amino group on the 5′ end of the aptamer) and, thus, the effective accessible affinity agent per unit area is higher compared to antibodies (*31*).

We used a DNA aptamer as the affinity agent (*K*_d_ = 5.8 nM) (*26*) targeting the ACE2 RBD in the S protein of the SARS-CoV-2 viral envelope. Because aptamers are chemically synthesized, they can be modified, and these modifications render them stable with little batch-to-batch variation (*32*). The aptamer contained a 3′ inverted deoxythymidine (*33*), making it stable in the presence of 3′ exonucleases and a 5′ amino linker and TEGylation that can extend half-life in biological samples and provide attachment to carboxy groups (*34*). The aptamer sequence and its secondary structures are presented in the electrospray ionization along with modifications used to allow it to be attached to a ─COOH group using 1-ethyl-3-(3-dimethylaminopropyl)carbodiimide (EDC)/*N*-hydroxysuccinimide (NHS) coupling chemistry that were present on the photocleavable (PC) linker attached to polymer surface (fig. S1 and table S1).

For the covalent attachment of the aptamer to the VP selection chip’s surface, we used a PC 7-amino coumarin heterobifunctional linker (Fig. 2G) (*28*). The PC linker is unique in its structure; it contained amino and carboxy termini to allow for two EDC/NHS reactions to (i) covalently attach the PC linker to the carboxylated COP surface and (ii) attach the aptamer containing a primary amine functionality at its 5′ end to the PC linker (*28*). The purity of the PC linker was tested via ultraperformance liquid chromatography–mass spectrometry and its ability to be cleaved following 2-min exposure to blue light (fig. S2).

We verified the ability of different blocking agents to minimize nonspecific adsorption to the surface of the VP selection chip that was activated with ultraviolet (UV)/O_3_ light. The blocking buffer contained 1% polyvinylpyrrolidone (PVP) and 0.5% bovine serum albumin (BSA) in phosphate-buffered saline (PBS) and showed low levels of nonspecific adsorption (1.4 ± 0.1%) of VPs to the selection chip’s surface compared to other blocking buffers tested (table S2). PVP/BSA was shuttled through the VP selection chip before sample processing but, after aptamer attachment to the PC linker, immobilized on chip’s surface.

### Surface plasmon resonance for determining VP/aptamer binding kinetics

**Fig. 3.**
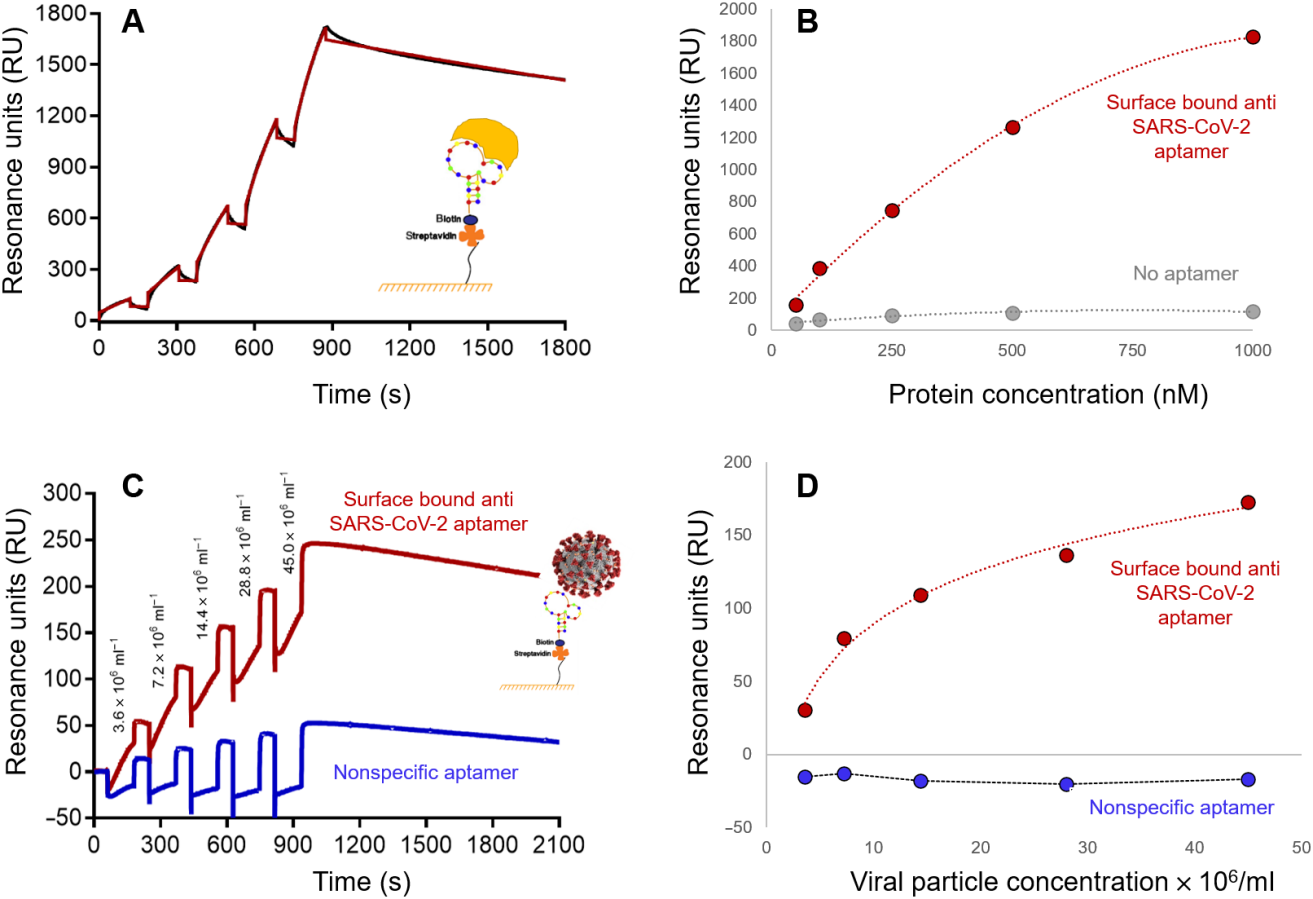
SPR of SARS-CoV-2 binding to affinity agent. (**A**) Sensogram showing the binding kinetics of recombinant SARS-CoV-2 S protein (RBD, rabbit Fc Tag, yellow object) to the SARS-CoV-2 aptamer. (**B**) Concentration isotherm for binding of the RBD S protein to its 51-nt aptamer. The control consisted of a channel with no aptamer. (**C**) Sensograms of SARS-CoV-2 VP binding kinetics to a specific (51-nt SARS-CoV-2) and nonspecific [human respiratory syncytial virus (HRSV)] aptamer. (**D**) Concentration isotherm of binding of heat-inactivated SARS-CoV-2 VP to its 51-nt aptamer. The control consisted of a channel with a random DNA sequence aptamer. The negative RU (blue line) is indicative of a negative bulk refractive index shift and lack of binding to the surface. The VP concentration varied between 3.6 × 10^6^ and 45 × 10^6^ genome equivalents of RNA per milliliter.

Figure 3A shows a typical sensogram for the RBD S protein of SARS-CoV-2 binding to the 51–nucleotide (nt) aptamer [for these experiments, the 51-nt aptamer was surface-bound to the surface plasmon resonance (SPR) flow cell], while the concentration dependence of the SPR signal from Fig. 3A is shown in Fig. 3B. *K*_d_ was determined to be 16.2 nM [association rate constant (*K*_a_) = 2.3 × 10^4^ M^−1^ s^−1^ and *K*_d_ = 2.7 × 10^−4^ s^−1^]. The reference-corrected sensograms for binding of the heat-inactivated SARS-CoV-2 particles to surface-bound aptamers are shown in Fig. 3C, and the concentration dependence of the SPR signal taken before each injection is plotted in Fig. 3D. As seen, the difference in resonance units (RU) and the shape of the binding curves between specific and nonspecific aptamers suggested that the heat-inactivated SARS-CoV-2 VPs associated with the 51-nt aptamer while no interactions were visible for the nonspecific aptamer. The VP-aptamer *K*_d_ was not calculated because of the heterogeneous nature of the virus. However, for two VOCs of SARS-CoV-2 (B.1.1.7 and B.1.351), the *K*_d_ values were found to be ~1 μM, ~100× higher than the data shown in Fig. 3 (C and D) indicating lower association compared to the original SARS-CoV-2 strain originating from Wuhan, China, for which the aptamer was originally designed (*26*).

### Analytical figures of merit of the VP selection chip

**Fig. 4.**
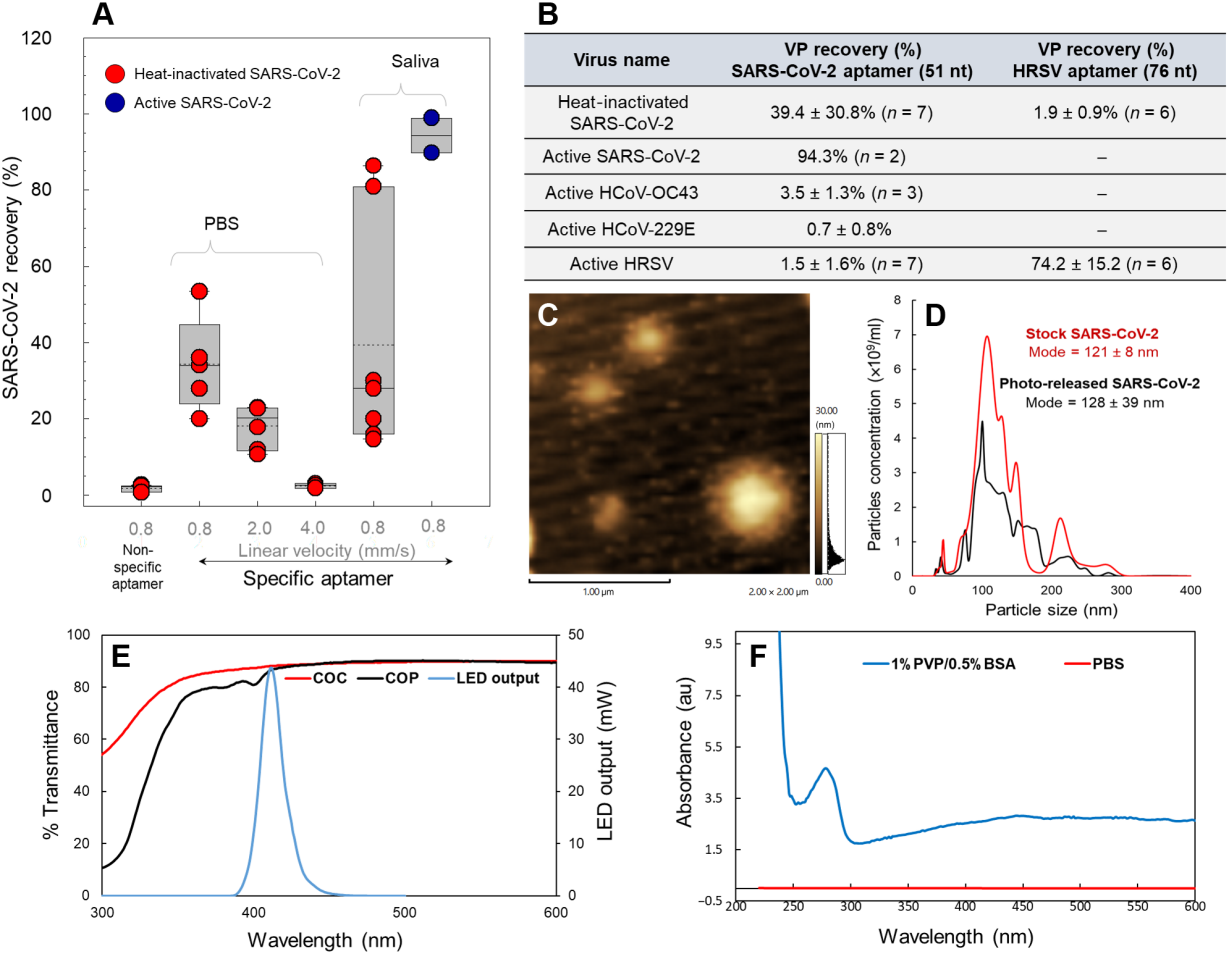
VP selection chip characterization and selection specificity. (**A**) Box plots representing SARS-CoV-2 and HRSV nonspecific binding to HRSV and SARS-CoV-2 aptamers, respectively, and recovery of VPs from buffer and saliva to their specific aptamers bound to the affinity bed at different linear flow velocities used for sample processing. (**B**) Summary of the recovery of different VPs to the VP selection chip using different aptamers. (**C**) AFM image of a selected and subsequently released SARS-CoV-2 particles using the VP selection chip. (**D**) NTA analysis of a stock solution of heat-inactivated SARS-CoV-2 (red trace) and selection and photo-released SARS-CoV-2 VPs from the chip (black trace). (**E**) UV-vis transmission spectra for COC (cyclic olefin copolymer) and COP plates (2 mm in thickness). LED output light range is shown as a reference. (**F**) Absorbance of a 1% PVP/0.5% BSA solution and PBS buffer in the UV-vis range. Absorbance spectrum measured in a 1-cm path length cuvette. au, arbitrary units.

Heat-inactivated SARS-CoV-2 VPs were seeded into PBS buffer and healthy donor saliva and infused through the VP selection chip to determine recovery. VPs were quantified via RT-qPCR in both the effluent (i.e., flow through) and following VP photo release (eluent), and based on mass balance, the recovery was assessed. RT-qPCR conditions, figures of merit, and primer/probe sequences can be found in fig. S3 and tables S3 and S4. The VP recovery from samples processed at different linear velocities (0.8 to 4.0 mm/s) is shown in Fig. 4A.

The SARS-CoV-2 seeding levels varied between 1 × 10^3^ copies/ml and 1 × 10^6^ copies/ml to represent typical VP loads in clinical samples (*35*). Specificity of the assay (i.e., VP cross-reactivity) was tested with the human respiratory syncytial virus (HRSV), human *Alphacoronavirus* 229E (HCoV 229E), and Betacoronavirus 1 (HCoV OC43). SARS-CoV-2 aptamer did not associate well with HCoV 229E and HCoV OC43 as the recovery was determined to be 0.7 and 3.5%, respectively (Fig. 4B). We should note that these human coronaviruses were not heat-inactivated as was the case for the SARS-CoV-2 VPs used in the previous studies. The SARS-CoV-2 aptamer showed no specific affinity for HRSV. In addition, SARS-CoV-2 VPs showed minimal affinity to the HRSV aptamer (Fig. 4B). However, the recovery of HRSV that was not heat-inactivated was found to be 74.2%.

The highest recovery of heat-inactivated VPs was observed at a linear sample flow rate of 0.8 mm/s (volume flow rate = 20 μl/min). It was observed that as the linear velocity of the infused sample decreased, the recovery of the VPs increased, which was attributed to a diffusion-controlled process for determining recovery. The recovery from saliva at a volumetric flow rate of 20 μl/min was determined to be 39.4% for heat-inactivated SARS-CoV-2. The recovery of the heat-inactivated VPs seeded into saliva was not statistically different from that found when the heat-inactivated SARS-CoV-2 was suspended in PBS (Fig. 4A).

Because heat inactivation of SARS-CoV-2 could affect aptamer binding to the epitope in the S protein due to possible denaturation, an experiment to determine the recovery of SARS-CoV-2 particles not subjected to heat inactivation was undertaken using a saliva sample from patients with COVID-19. A self-referencing method was used for these studies (*36*), which used three VP selection chips connected in series with the saliva sample flowed through the chips at 20 μl/min. Following sample infusion, SARS-CoV-2 particles isolated from each chip in the series were quantified using RT-qPCR from which the recovery could be calculated (*36*). The recovery of native SARS-CoV-2 using the 51-nt aptamer and VP selection chip was found to be 94.7 ± 7%. The recovery of the B.1.1.7 VOC spiked into healthy unvaccinated donor’s saliva was 35 ± 12%.

VPs were characterized via atomic force microscopy (AFM) to visually confirm that following selection and release, the particles retained their typical morphology (i.e., spherical shape with a halo produced by the S protein), and nanoparticle tracking analysis (NTA), which was used to determine the size of the VPs and estimate concentration. Representative data are shown in Fig. 4 (C and D). For AFM analysis, VPs were photo-released in water, and 2 μl was deposited onto a clean Si wafer and dried at ambient temperature before imaging. Inspection of AFM images indicated that most of the selected and released VPs were <250 nm in diameter (fig. S4A). Appearance of a larger size of some VPs (Fig. 4C) may result from flattening of the particle when drying on the Si surface (the height of the VPs was only ~30 nm and suggestive of flattening of VPs). We also visualized the captured and released VPs using transmission electron microscopy (TEM) (fig. S5). VP sizes ranged from 20 to 70 nm; however, this smaller size may be attributed to VP dehydration during sample preparation. NTA results are presented in Fig. 4D for the stock solution of heat-inactivated SARS-CoV-2 VPs and those selected and released using the microchip, which indicated a mean diameter of 133 ± 16 nm and 138 ± 27 nm, respectively, with no statistical difference noted. NTA was not used to secure SARS-CoV-2 quantitative information owing to large errors associated with these measurements in terms of particle concentration. Quantitative information was thus secured using RT-qPCR assay because of its highly quantitative nature.

We assessed the PC release efficiency of aptamer-selected VPs. Release efficiency was calculated from the ratio of released VPs at 2 min with respect to all VPs released after 10 min of blue light exposure. The release efficiency was determined to be 88 ± 10% (*n* = 10) for SARS-CoV-2 after 2-min exposure using an energy of 41 kJ/s·m^2^ (λ = 412 nm; 32 ± 4 mW/cm^2^). The UV-visible (UV-vis) absorbance of COP was assessed as well to ensure that 412-nm light was able to be transmitted through the substrate and cover plate of the plastic chip. The results shown in Fig. 4E demonstrated that COP transmitted 85% of the blue light and PBS did not absorb in this wavelength range (Fig. 4F).

Free gRNA (RNA not encased within the viral envelope) adsorption onto the VP selection chip’s surface was also assessed. gRNA isolated from SARS-CoV-2 VPs was passed through the VP selection chip at 0.8 mm/s (20 μl/min) containing the surface-attached 51-nt aptamer. Following sample infusion, the chip was washed and subjected to 2-min blue light exposure to cleave the PC linker, and the eluent was tested via RT-qPCR. Less than 0.3% of gRNA was detected by RT-qPCR (fig. S4B). We also confirmed the integrity of the gRNA following VP isolation, release, and lysis using gel electrophoresis. A representative electropherogram is shown in fig. S6. Most of the gRNA was in the form of a full-length 30-knt gRNA.

### SARS-CoV-2 selection from saliva samples

**Fig. 5.**
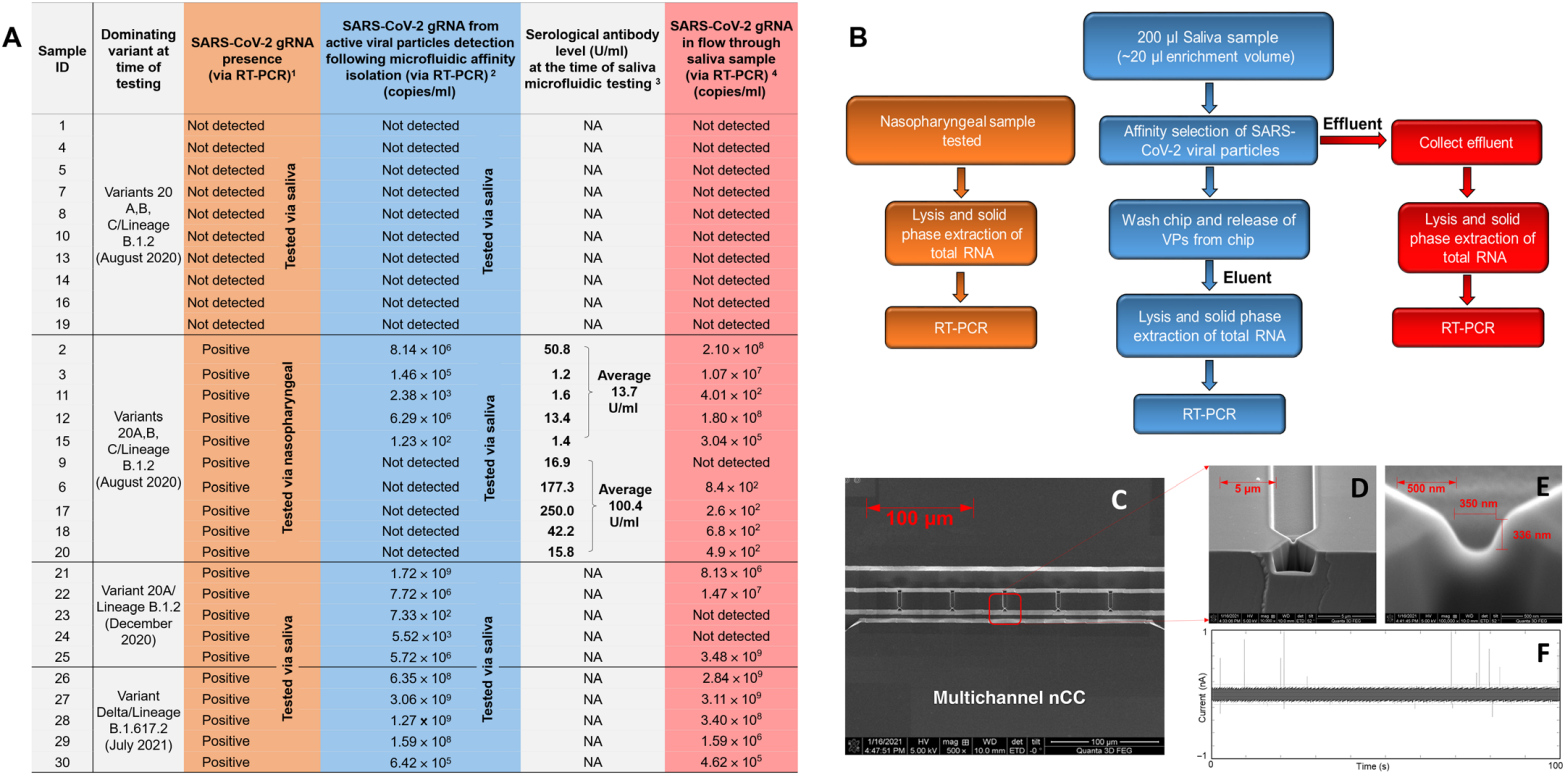
VP selection assay for the analysis of clinical samples. (**A**) Summary of results for saliva samples secured from anonymous donors. (^1^) Healthy donors test were performed by Sinochips Diagnostics with 0.2 ml of saliva sample. COVID-19–positive individuals’ status was confirmed using the Cepheid Xpress SARS-CoV-2 test from nasopharyngeal swabs. (^2^) A saliva sample of 0.2 ml was processed using the 51-nt SARS-CoV-2 aptamer–modified SARS-CoV-2 VP selection chip. Ten microliters of the photo-released VPs were evaluated by RT-qPCR. *N1* and/or *N2* are virus nucleocapsid (*N*) gene fragments targeted for specific detection of SARS-CoV-2 VPs. (^3^) A Roche-based antibody test was performed at Sinochips Diagnostics, Olathe, KS. (^4^) Approximately 0.2 ml of the saliva flow through from the microfluidic chip was evaluated by RT-qPCR. *N1* and/or *N2* are virus nucleocapsid (*N*) gene fragments targeted for specific detection of SARS-CoV-2. (^5^) Approximately 20 μl of the effluent was heat-inactivated. (**B**) Experimental design for evaluating the 30 clinical samples received for this study. Matched nasopharyngeal and saliva samples were secured for each of the 30 patients through an IRB-approved protocol at the University of Kansas Medical Center. (**C**) SEM of the five–in-plane nanopore focused ion beam milled into a Si wafer. (**D**) SEM showing a bridge channel flanking the access microchannels. The in-plane nanopore is positioned at the input side of the bridge channel shown in the figure. The SEM shown here is the plastic chip made in COP that was fabricated by imprinting. (**E**) High-resolution SEM showing an in-plane nanopore imprinted into a COP plastic chip, which was designed to have an approximate 350-nm effective diameter. (**F**) nCC transient current traces for heat-inactivated SARS-CoV-2 VPs.

We collected 30 saliva samples through an Institutional Review Board (IRB)–approved protocol at the University of Kansas Medical Center (table S5). Each sample was tested in an approved COVID-19 testing center using RT-qPCR (FDA-approved protocol). The remaining de-identified samples were then shipped to the University of Kansas, Lawrence, and stored at −80°C until required for testing. To evaluate the ability to distinguish samples with active versus nonactive disease (active disease being define as a sample containing intact SARS-CoV-2 VPs with an accessible ACE2 RBD of the S protein), we followed the scheme shown in Fig. 5. The orange column in Fig. 5A and the orange boxes in Fig. 5B represent the testing strategy for potential patients with COVID-19 carried out at the certified testing laboratory. This strategy effectively identified the presence of viral gRNA in the sample, but because all samples were subjected to lysis of the VPs and extraction of gRNA from the lysate, it could not identify the source of the gRNA. For columns 4 and 6 (i.e., blue and red boxes) in Fig. 5 (A and B), saliva samples were passed through a VP selection chip, blue light released from the chip following selection, and subjected to RT-qPCR. In this case, the eluent can contain intact SARS-CoV-2 particles with an accessible epitope in the S protein for aptamer binding (free gRNA contamination of <0.3% for the VP selection chip; see fig. S4B), as confirmed by RT-qPCR with primers specific to SARS-CoV-2. Column 6 in Fig. 5A (red boxes) represents data for the VP selection chip’s effluent, and successful RT-qPCR results would represent amplification of free gRNA and/or unselected SARS-CoV-2 particles due to an inaccessible S protein RBD epitope.

The saliva samples were processed using the VP selection chip (total input sample volume = 200 μl; effluent). Following washing of the VP selection chip, the chip was exposed to blue light for the release of selected VPs (i.e., eluent). The eluent and effluent were then subjected to RT-qPCR analysis. The results of this study are summarized in Fig. 5A. The samples deemed “not detected” by RT-qPCR in the CLIA (Clinical Laboratory Improvement Amendments)-approved and CAP (College of American Pathologists)-accredited testing laboratory agreed 100% with our results secured following VP selection chip processing of the saliva sample (clinical specificity = 100%). In the case of the first 10 positive COVID-19 samples (as determined by the certified clinical laboratory RT-qPCR analyses) collected in August 2020, 5 samples were found to be positive for the presence of SARS-CoV-2 VPs when the eluent from the VP selection chip was evaluated via RT-qPCR. On the basis of RT-qPCR results of the effluent, all samples but one tested positive (Fig. 5A). For sample #20, we processed a larger volume (400 μl) of saliva, and VPs were still not detected by RT-qPCR. To test for a negative matrix effect of saliva for which we could not detect VPs in the chip’s eluent, we pooled saliva from patients #6 (negative) with #2 (positive) and #18 (negative) with #11 (positive). In these pooled samples, VPs were detected, therefore, rejecting the supposition that the saliva matrix from patients #6 and #18 affected the results.

For the saliva samples collected in August 2020, the VP selection chip coupled with RT-qPCR detected active VPs ranging between 1.23 × 10^2^/ml and 8.14 × 10^6^/ml. Negative RT-qPCR results in the eluent for five samples (#6, #9, #17, #18, and #20) suggested the absence of active VPs in the saliva above the limit of detection of our RT-qPCR assay [threshold cycle (Ct) ≤ 40]. In patients with undetected active VPs, the average neutralizing antibody concentration was 7× higher (100.4 U/ml; Fig. 5A) than the antibody concentration found in the plasma of patients in which active SARS-CoV-2 particles were found (13.7 U/ml).

During the summer of 2020, when these saliva samples were collected, the dominant variants in the United States were 20A,B,C (lineage B.1.2). These clades originated in the southern region of the United States and acquired a D614G mutation and a Q677H mutation that is adjacent to the furin cleavage site of the non-RBD of the S protein (*37*). The Q677H mutation has been found in other SARS-CoV-2 lineages as well. This mutation, however, falls outside of S protein RBD that was the epitope of the 51-nt aptamer used here (i.e., S protein fragment spanning Arg^319^-Phe^541^; RBD, YP_009724390.1) and, thus, is less likely to affect the antigen/aptamer binding.

In December 2020 (www.gisaid.org/phylodynamics/global/nextstrain) (*38*), most of samples collected in eastern KS and western MO, which is the source of the samples analyzed in this study, also showed the presence of the 20A clade of SARS-CoV-2 (Fig. 1B). In all five saliva samples collected in December 2020 (#21 to #25), the VP selection chip detected active VPs. In two samples, no gRNA was detected in the effluent (#23 and #24). On average, viral loads of these saliva samples were higher than samples collected in August 2020 (3.5 × 10^8^/ml versus 2.9 × 10^6^/ml).

Saliva samples collected during summer 2021 were also analyzed using the VP selection chip. On the basis of sequencing data (www.gisaid.org/phylodynamics/global/nextstrain) (*38*), the most frequently detected SARS-CoV-2 clade in KS and MO at that time was the Delta variant (Fig. 1B). The Delta variant has two mutations in the non-RBD, D614G and P681R. A P681R substitution makes the protein sequence more accommodating for furin to cut effectively, allowing ~75% of S proteins to be cleaved that facilitates virus entrance into an epithelial cell bearing an ACE2 receptor (*39*). Three mutations identified in the RBD region of the Delta VOC are K417N associated with conformational changes in the S protein, and L452R and T478K that have been shown to increase affinity binding to ACE2 receptors and create steric hindrance allowing immune escape (*39*). While these mutations can affect epitope orientation causing immune escape from large antibodies (150 kDa), smaller molecules such as aptamers (≤16 kDa) may be less affected by epitope orientation. Quantification of the eluent from the VP selection chip for five saliva samples collected in the summer of 2021 demonstrated very high viral loads (1.02 × 10^9^/ml) of intact SARS-CoV-2 VPs. These results suggest that the Delta variant was affinity selected using the 51-nt aptamer.

## DISCUSSION

A recent survey of the general public, scientists, engineers, and health professionals highlighted several important needs for evolving COVID-19 technologies (*40*): (i) development of a point-of-care screening test for COVID-19; (ii) diagnose highly contagious individuals; (iii) develop a noninvasive, quick, inexpensive, and effective test that people can do themselves; and (iv) identify who needs COVID-19 testing in people with chronic conditions.

While RT-qPCR and rapid antigen tests have been the cornerstone of COVID-19 testing, they have the inability to distinguish patients with active disease that may be considered to be infectious and those that do not have the capacity to infect others. PCR-based assays may overestimate the number of people actively spreading disease as they are unable to distinguish between active and nonactive disease because the RNA extraction procedure does not determine the source of the RNA giving a positive test. For those that can be considered infectious or have active disease, there are several criteria including (i) intact VPs that contain gRNA, (ii) an accessible RBD on the S1 subunit to allow binding of the VP to the ACE2 receptor of the host to transfer its gRNA to cells to allow for replication, and (iii) high viral load to accommodate the low take rate for replication (*41*). The inclusion of the VP selection chip decorated with the surface-bound 51-nt aptamer to preprocess samples before RT-qPCR generates an eluent that only contains active SARS-CoV-2 VPs and when coupled to RT-qPCR, can provide information as to the viral load of those with active disease and, thus, fulfill the three aforementioned criteria for determining the infectious status of a patient with COVID-19.

The importance of the ability to discriminate between active and nonactive disease is paramount because it can determine the length of time needed in quarantine or help planning mass quarantines; extended quarantines can have serious socioeconomic consequences, post-traumatic stress symptoms arising from financial loss, stigma, boredom, or fear of infection (*42*). The fears of infection and/or extended quarantines have created delayed diagnosis of other serious diseases because annual checkups are either deferred or canceled. A population-based study indicated that because of delayed diagnosis during pandemic, cancer-related deaths will substantially increase over the next 5-year period (*43*). Reducing quarantine times and/or reducing infection fears can improve the outcome for non–COVID-19 mortalities.

A report on the temporal dynamics of viral shedding and transmissibility of COVID-19 indicated that 44% of transmission occurred in a presymptomatic stage of the viral load profile (*44*). Thus, PCR testing alone, despite its high sensitivity and specificity, may elongate quarantine times because of the inability to determine the source of gRNA used for the measurement. Patients can show long-term positive RT-qPCR results arising from persistent viral RNA shedding long after infectivity has ceased (*45*, *46*). Median time for the SARS-CoV-2 gRNA persistent presence was determined to be 54 days (*47*). It has also been shown that SARS-CoV-2 immunoglobulin M (IgM) and IgG levels were persistently high 56 days after infection (*44*).

While viral infectivity can be deduced from culturing, its extensive workflow and long result turnaround time and the need for BSL-3 (biosafety level 3) facilities makes this approach intractable for determining infectivity for clinical testing or screening (*48*). In addition, challenges with this approach are that determining exact symptom onset is difficult in asymptomatic individuals.

We outlined a unique technology that addresses the aforementioned challenges and is based on the ability to affinity select SARS-CoV-2 particles from clinical samples and confirm their presence using RT-qPCR. The technology consists of a simple microfluidic device for the affinity selection of VPs using an aptamer (see Fig. 2), which is directed against the ACE2 RBD of the S protein. The design and performance criteria of the VP selection chip were (i) short processing time (<20 min); (ii) large dynamic range to accommodate the range of VPs that can be found in clinical samples (500 to 10^8^ VPs/ml) (*49*); (iii) chip manufactured at high production rates, low cost, and with tight compliancy for screening applications that demand disposable devices (*29*); and (iv) a stable affinity agent that accommodates long-term storage to allow stockpiling for future pandemics. The important attributes of our technology satisfies two of four needs highlighted from the 83-person survey cited above (*40*). To accommodate the other two needs, which includes point-of-care screening and a simple-to-use technology, it will be necessary to remove RT-qPCR from the counting phase of the measurement and replace with a simple particle enumeration strategy.

While antibodies have been widely used for the affinity selection of biological targets, aptamers as recognition elements offer several valuable qualities. The aptamer used in our technology showed a recovery for non–heat-inactivated SARS-CoV-2 of 94.3% with minimal binding affinity to other human coronaviruses (see Fig. 4B). We note that the recovery for the SARS-CoV-2 VPs was nearly 10% higher than we observed for antibody-based affinity isolation of CD8-bearing extracellular vesicles using the same type of chip and operating conditions (*30*). While aptamers exhibit notable affinity to their targets with *K*_d_ values ranging from the high picomolar to low nanomolar range (*50*), they also have the ability to place functional groups at well-defined locations within their structures for surface attachment that result in highly ordered orientations on surfaces (i.e., amino group at 5′ end of the aptamer in our case) keeping nearly 100% of the immobilized aptamers accessible that can improve recovery of targets. On the other hand, antibodies are randomly attached to solid surfaces resulting in only a small percentage of the immobilized antibody available for binding to antigens, which can affect recovery of the solution-born target (*31*). In addition, DNA aptamers, as used herein, are particularly stable and can be stored for extended periods of time without cold storage.

In our study, once the aptamers were covalently attached to the surface of the VP selection chip, no reagents were required to carry out the selection phase of the measurement, except for common buffers such as PBS. This was facilitated by the blue light release of the selected VPs from the chip’ surface eliminating the need for additional reagents (*28*). The 51-nt aptamer, which was generated from the original SARS-CoV-2 clad, still showed affinity for the B.1.1.7 variant, despite structural modifications in the ACE2 RBD of the S protein, albeit with a lower binding affinity compared to the original SARS-CoV-2 clad. High binding affinity aptamers and/or selection specificity for the specific VOCs of SARS-CoV-2 can be generated using an in vitro SELEX (systematic evolution of ligands by exponential enrichment) process to allow for strain-specific identification. Understanding the identity of the particular VOC infection irrespective of vaccination status can be important when several VOCs coexist within certain geographical regions (Fig. 1B). For example, the three EUA-approved vaccines for COVID-19 were generated against the wild-type SARS-CoV-2 S protein originating from Wuhan, China. The neutralizing potency of these vaccines for the Omicron variant was found to be reduced compared to the Delta variant and the wild-type but demonstrated notable neutralizing potency for those that received the booster (*51*).

Figure 5 offers some interesting results and conclusions for using the VP selection chip with RT-qPCR compared to RT-qPCR alone for analyzing gRNA. In August 2020, of the 10 samples that were positive by conventional RT-qPCR, 5 were found to be positive for active disease when the eluent of the VP selection chip was analyzed by RT-qPCR. However, inspection of the results from the effluent showed 90% of those samples to be positive by RT-qPCR. This indicated that five of the nine positive samples contained active VPs with an intact virus envelope and an S protein with accessible RBDs targeting the ACE2 receptor. The effluent that tested positive using RT-qPCR may contain free gRNA fragments and/or VPs with no accessible ACE2 RBD, while the eluent (i.e., photo-released fraction) consisted only of intact VPs with an accessible ACE2 RBD in the S protein (see fig. S4). We should also note that the photo release of affinity-captured VPs assists in assuring that the chip’s eluent contains only affinity-selected VPs because nonspecifically adsorbed VPs are not released from the surface using blue light; this adds an additional specificity level to the reported assay.

Data shown in Fig. 5A for samples that did not show the presence of active VPs, a higher concentration of neutralizing antibodies, which could be indicative of the immune system’s ability to disable the SARS-CoV-2 VPs by blocking the S protein binding to the ACE2 receptor (*52*), rendering it inactive and, therefore, unable to be affinity selected by the 51-nt aptamer used in the VP selection chip. Recent work has demonstrated that the development of neutralizing antibodies after COVID-19 symptom onset correlated with viral control (i.e., clearance of virus due to blocking of S antigens or nucleoproteins to counter an infection and viral replication) (*53*).

Screening tests, such as those geared for at-home use, for SARS-CoV-2 infections can be an essential tool for effective containment of COVID-19 or other infectious diseases because it allows for more frequent testing and provides rapid results compared to clinical testing performed less frequently as these tests require a centralized laboratory (see Fig. 1A). While new at-home technologies based on PCR or antibodies are evolving, they cannot determine the infectivity status of the patient, only if infection has occurred or not. To address these challenges, we presented a technology to identify patients who have been infected with SARS-CoV-2 and carry active VPs. The microfluidic device, which were made from a thermoplastic, can be fabricated by injection molding that is conducive to high-scale production at low cost appropriate for large-scale screening (*29*).

Testing for COVID-19 in saliva offers advantages. Self-collected saliva sampling can be performed with no supervision from health care providers (*54*) and eliminate the risk of health care worker infection (*55*). In addition, saliva samples for COVID-19 testing have demonstrated comparable results to nasopharyngeal and oropharyngeal samples (*54*–*56*). It has been found that viral loads from saliva averaged 3.6 × 10^6^ VPs/ml (range = 9.9 × 10^2^ to 1.8 × 10^8^ particles/ml) (*44*).

The use of saliva for COVID-19 testing as demonstrated here (see Fig. 5A) in conjunction with the VP selection chip can provide a venue for simple at-home testing whether the RT-qPCR phase of the assay can be replaced with a simple particle enumeration method, satisfying all of the needs required for COVID-19 testing (*40*). We are currently working on a label-free enumeration chip of affinity-selected VPs to replace the RT-qPCR readout step used here. The label-free enumeration strategy is performed by a plastic chip containing an extended nano-Coulter counter (XnCC) (Fig. 5, C to E). The XnCC contains five-narrow constrictions (~350-nm effective diameter) that can detect single VPs via resistive pulse sensing (RPS) (*57*). Unique to this device is the ability to place five pores in parallel to increase sampling throughput reducing processing time and also increase sampling efficiency that results in improvements in the concentration limit of detection. The chips are fabricated in a thermoplastic so that they can be produced in a high production mode and at low cost using injection molding. Figure 5F shows RPS current traces for heat-inactivated SARS-CoV-2 particles suspended in PBS and passing through the XnCC chip. The analytical figures of merit of this XnCC chip in conjunction with the VP selection chip will be reported in a subsequent manuscript. Other label-free methods could be considered as well, such as SPR. However, SPR instrument and consumable costs are high, and the processing steps are not amenable to POCT. In addition, SPR has a limited dynamic range.

We are also developing new aptamers that have unique characteristics, such as the ability to show large differences in binding to subtle molecular changes within the S protein and high binding affinities to the epitope for which they are directed against. For example, the smaller size of aptamers compared to antibodies can allow for properly designed aptamers to query minor structural variations that may not be accessible to antibodies based on size considerations. One characteristic feature of the SARS-CoV-2 S protein is its trimeric structure. Therefore, multivalent interactions can be used to improve the *K*_d_ of the target/aptamer association. For example, self-assembled homotrivalent affinity agents directed against a target of interest with low picomolar binding affinities can be quickly generated from monomeric affinity agents that have 100- to 1000-fold larger *K*_d_ values (*58*).

## MATERIALS AND METHODS

### Experimental design

The primary objective of this study was to demonstrate the use of a microfluidic-bound aptamer for the efficient selection of active SARS-CoV-2 particles found in saliva samples of patients with COVID-19. The microfluidic provided a defined source of gRNA and when enumerated via RT-qPCR could determine the infectivity status of the patient.

### Model VPs for determining the assay’s analytical figures of merit

For determining the analytical figures of merit of the VP selection chip, heat-inactivated SARS-CoV-2 [American Type Culture Collection (ATCC), VR-1986HK] VPs were used. SARS-CoV-2 VPs were inactivated at 65°C for 30 min making it unable to replicate. HRSV (strain A2, ATCC, VR-1540) was also used in these studies to demonstrate the VP selection chip’s ability to be reprogrammed for other VPs. HRSV was affinity selected using an aptamer identified by Percze *et al.* (*59*). HCoV OC43 (ATCC, VR-1558), HCoV 229E (ATCC, VR-740), and HRSV, which were all active, were used for specificity studies with the SARS-CoV-2 aptamer–modified VP selection chip. All experiments were performed in a BSL-2 laboratory.

### VP selection chip fabrication

The VP selection chips used in these studies were fabricated in COP via injection molding (Stratec, Austria) using a mold insert made via UV-LiGA (*60*). Figure 1D provides rapid scanning confocal images of a selection bed and the dimensional features of the pillars contained in one of the seven selection beds. As can be seen, the chip consisted of pillars used to increase the available surface area to accommodate high loads of VPs without significantly increasing the footprint of the chip and reducing the diffusional distances to allow for frequent interactions with surface immobilized aptamers that resulted in high recovery of VPs.

### Aptamer for SARS-CoV-2 selection

The DNA aptamer was designed against the S protein by Song *et al.* (*26*) using an ACE2 competition assay and a machine learning screening algorithm (see fig. S1).

### Surface immobilization of PC linker and aptamer

The structure of the PC linker (5-((7-((2-(2-(2-aminoethoxy)ethoxy)ethyl)(ethyl)amino)-2-oxo-2*H*-chromen-4-yl)methoxy)-5-oxo-pentanoic acid) is shown in fig. S2. Detailed synthetic routes and characterization of the PC linker has been described elsewhere (*28*) and summarized in fig. S2.

### SPR of VPs and DNA aptamer

SPR was performed using a BIAcore T-200 instrument with a CMD200L chip (Xantec Bioanalytics, GmbH; Dusseldorf, Germany). Data were collected at 25°C using a flow rate of 20 μl/min and a running buffer of 10 mM Hepes/140 mM NaCl/0.005% (m/v) Tween 20. NaOH (10 mM) was used for regeneration of the surface. A ligand-capture approach was used to assess interactions of purified proteins and VPs to surface-bound aptamers. Briefly, all flow cells of a CMD200L sensor chip were activated by NHS/EDC, and each was further modified by injection of Neutravidin (Thermo Fisher Scientific). A reference surface was prepared by blocking with 10 μM biotin, while experimental surfaces were prepared by injecting 100 nM biotinylated aptamers (either aptamer designed for SARS-CoV-2 or aptamer for HRSV as a nonspecific control) into separate SPR flow cells. A recombinant form of the SARS-CoV-2 S protein (accession number YP_009724390.1, Arg^319^-Phe^541^) fused to the Fc region of rabbit IgG1 at its C terminus [molecular weight (*M*_w_) = 50.3 kDa] was used as the analyte for initial studies. SPR experiments were performed in a single-cycle mode across all four flow cells using reference subtraction. Sequential 2-min injections of increasing S protein concentrations (50, 100, 250, 500, and 1000 nM) were followed by a final dissociation phase of 20 min. The association and dissociation rate constants (*K*_a_ and *K*_d_, respectively) and equilibrium *K*_d_ values for S protein fusion binding to the aptamer were calculated assuming a 1:1 Langmuir model using BiaCore T-200 Evaluation software. Sensograms corresponding to binding of SARS-CoV-2 VPs (3 × 10^6^ to 5 × 10^7^ VPs/ml) to specific (51-nt SARS-CoV-2) and nonspecific (HRSV) aptamers were also obtained (Fig. 3D). In these experiments, the concentration-dependent signal for binding of heat-inactivated SARS-CoV-2 VP to the SARS-CoV-2 aptamer was compared to the signal from a control flow cell modified with a random DNA sequence aptamer. Specific signal was obtained by subtracting the respective reference-corrected sensogram series using GraphPad Prism.

### Statistical analysis

All data were analyzed by calculating averages, modes, and SDs. No other statistical methods were used.

### VP selection chip sample processing

Saliva samples were hydrodynamically driven through the VP selection chip using a syringe pump (New Era Pump Systems Inc., Farmingdale, NY USA) and a 1-ml tuberculin syringe fitted with a capillary connector (Inner-Lok union capillary connectors; Polymicro Technologies) and barbed socket Luer Lock fittings (3/32″ ID, McMaster-Carr). Saliva samples were centrifuged at 1000*g* for 5 min to pellet buccal cells. For all saliva samples analyzed, none were diluted and no chip failure was noticed. Samples were infused into the device at varying volumetric flow rates (20 to 100 μl/min). Following sample introduction, the VP selection chip was rinsed with PBS at 50 μl/min. All buffer solutions were filtered through a 0.45-μm polypropylene filter (Thermo Fisher Scientific) before use. VPs were photoreleased in ~20 to 35 μl of PBS and enumerated using RT-qPCR.

### Reverse transcription quantitative polymerase chain reaction

Samples were first subjected to RNA extraction using a Zymo viral RNA extraction kit following the manufacturer’s protocol. Purified total RNA was eluted in ~8 μl of nuclease free water. RT-qPCR was used as the standard method for assay optimization, validation, and VP enumeration. cDNA was synthesized via RT with random primers using ProtoScript II First Strand cDNA Synthesis Kit (NEB) according to the manufacturer’s instructions. Universal PCR supermix was obtained from Bio-Rad. Clinical samples were evaluated with RT-qPCR (iTaq Universal Probes One-Step Kit).

### Healthy donor and COVID-19 patient testing

Informed written consent was obtained from all individual participants included in the study. Patients were consented either at the KU Hospital as part of a treatment clinical trial by a study member on the IRB-approved protocol through the inpatient setting or by the Biospecimen Repository Core Facility staff at the KU Medical Center, the KU Hospital, or the Kansas Wyandotte Health Department’s COVID-19 screening site. De-identified specimens and their accompanying clinical data were handled in an anonymous (coded) fashion. All samples were evaluated by Sinochips Diagnostics (Olathe, KS). Two hundred microliters of saliva was eluted into 50 μl using the MagMAX Viral/Pathogen Nucleic Acid Isolation Kit (Applied Biosystems, catalog no. A42352) and the KingFisher Flex system (Thermo Fisher Scientific). The eluate (5 μl) was analyzed using the COVID-19 Nucleic Acid RT-qPCR Test (ZhuHai Sinochips Bioscience Co. Ltd., EUA201020) on an Applied Biosystems ABI 7500 Fast Dx Real-Time PCR system with SDS software v.1.4.1. Patients with COVID-19 were tested at a CLIA-approved KU Health System Laboratory upon admission to the hospital. Testing was performed using a Cepheid Xpress SARS-CoV-2 instrument with sample consisting of a nasopharyngeal swab and were collected according to the manufacturer’s protocol under an FDA EUA. Informed consented non–KU Health System patients collected during screening via the Wyandotte Health System, KS, were also tested by Sinochips Diagnostics using EUA-approved clinical tests. De-identified saliva samples were sent to the University of Kansas (Soper laboratory) for testing using the microfluidic chip in a blinded fashion. These samples were selected by a study coordinator and, thus, do not represent the positivity rate of COVID-19 testing.

## Supplementary Materials

### This PDF file includes:

Supplementary materials and methods Figs. S1 to S6 Tables S1 to S5 References

## Notes

View/request a protocol for this paper from *Bio-protocol*.
