# Supplementary material for: Microfluidic affinity selection of active SARS-CoV-2 virus particles
Source: Sci Adv. 2022 Sep 28;8(39):eabn9665. doi: 10.1126/sciadv.abn9665 (PMC9519043; doi:10.1126/sciadv.abn9665)
Supplement: Supplementary file 1 — Supplementary materials and methods Figs. S1 to S6 Tables S1 to S5 References [file sciadv.abn9665_sm.pdf]

Science Advances
AAAS

Supplementary Materials for

Microfluidic affinity selection of active SARS-CoV-2 virus particles

Sachindra S. T. Gamage *et al.*

Corresponding author: Andrew K. Godwin, agodwin@kumc.edu; Malgorzata A. Witek, mwitek@ku.edu; Steven A. Soper, ssoper@ku.edu

Sci. Adv. **8**, eabn9665 (2022)
DOI: 10.1126/sciadv.abn9665

This PDF file includes:

Supplementary materials and methods
Figs. S1 to S6
Tables S1 to S5
References

Materials and chemicals. Reagent grade IPA (isopropyl alcohol), 1-ethyl-3-[3-dimethylamino-propyl] carbodimide hydrochloride (EDC), N-hydroxysuccinimide (NHS), anhydrous acetonitrile (ACN), anhydrous dimethylformamide (DMF), anhydrous dichloromethane (DCM), anhydrous triethyl amine (TEA), bovine serum albumin (BSA), Polyvinylpyrrolidone – 40 kDa (PVP-40), and polyethylene glycol (PEG) were all secured from Sigma-Aldrich and used as received. Nuclease free water was obtained from Fisher Scientific, and Virkon S was purchased from Dupont. Phosphate buffered saline (PBS, pH = 7.4) was purchased from Gibco Laboratories. Universal PCR and iTag supermixes were obtained from Bio-Rad. Other reagents included TapeStation supplies (Agilent), Zymo Viral RNA isolation kit (Zymo Research), and ProtoScript II First Strand cDNA Synthesis Kit (New England BioLabs). Aptamers, primers, and probes were received from Integrated DNA Technologies.

Model virus particles (VPs) for determining the analytical figures-of-merit of the VP selection chip. For determining the analytical figures-of-merit of the VP selection chip, we employed heat inactivated SARS-CoV-2 (ATCC, VR-1986HK). SARS-CoV-2 VPs were inactivated at 65°C for 30 min making it unable to replicate. Human Respiratory Syncytial Virus (HRSV, strain A2, ATCC, VR-1540) was also used in these studies to demonstrate the VP selection chip's ability to be reprogrammed for other VPs. HRSV was affinity selected using an aptamer identified by Percze *et al*. *(59)*. HCoV OC43 (ATCC, VR-1558), HCoV 229E (ATCC, VR-740), and HRSV, which were not heat inactivated, were used for specificity studies with the SARS-CoV-2 aptamer-modified VP selection chip. All experiments were performed in a BSL-2 laboratory.

A

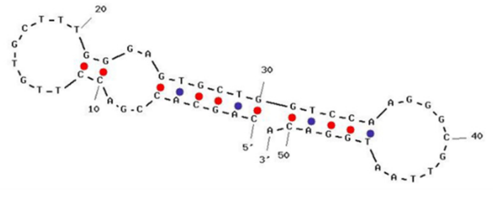

B

Aptamer	Length (nt)	ΔG (kcal/mole)	Tm(°C)	ΔH (kcal/mole)	ΔS (cal/K x mole)
SARS-CoV-2	51	-7.4	47.6	-104.9	-327.1

C

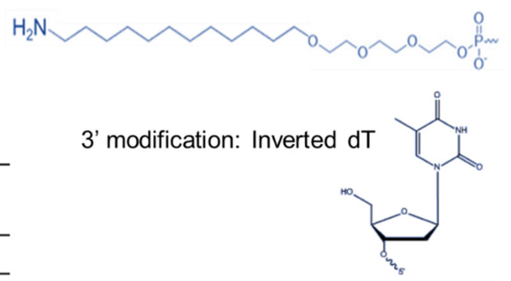

Figure S1. Aptamer structure and 3' modification. (**A**) Structure of the aptamer for SARS-CoV-2 *(26)*. (**B**) Thermodynamics showing the Gibbs free energy, enthalpy, and entropy for the most stable secondary structure of the 51 nt SARS-CoV-2 aptamer. (C) Functional groups employed within the aptamer structure to allow for covalent attachment of the aptamer to an UV/O_3 activated plastic surface (5' end with C12 linker and primary amine) and stabilization of the aptamer (3' end with inverted dT residue). Shown here is the secondary structure analysis for the 51 nt SARS-CoV-2 aptamer only.

Aptamer for SARS-CoV-2 selection. The DNA aptamer was designed against the receptor binding domain (RBD) of the SARS-CoV-2 S protein. Song *et al. (26)* used ACE2 competition and a machine learning screening algorithm to develop the aptamer used herein (**Figure S1**).

The most stable secondary structure of the 51 nt SARS-CoV-2 aptamer is shown in **Figure S1A** with thermodynamic properties shown in **Figure S1B**. Secondary structures were determined using UNAFold/OligoAnalyzer software (Integrated DNA Technologies) and was based on free energy minimization. The aptamer contained a primary amine with a C12 linker and TEG spacer (spacer 9) at its 5' end and an inverted dT at its 3' end (**Figure S1C**). The affinity of the aptamer has been reported to be 5.8 nM, which is smaller than the reported K_d of ACE2 and the SARS-CoV-2 S protein (34.6 nM) *(18)*. Sequences of all the aptamers used in these studies are listed in **Table S1**.

Table S1. Sequences of the aptamers used in these studies.

Virus Name	Aptamer Target Proteins	DNA Aptamer Sequence 5'-3'
SARS-CoV-2 (ATCC® VR-1986HK™ (heat inactivated)	S (spike)	CAGCACCGACCTTGTGCTTTGGGAGTGCTGGTCCAA G GGCGTTAATGGACA<br>K_d =5.8 ± 0.8 nM
Human respiratory syncytial virus (HRSV) type A2 ATCC® VR-1540™	glycoprotein (G)	TAG GGA AGA GAA GGA CAT ATG AT AGT GCG GTG AGC CGT CGG ACA TAC AAA TAC TT GAC TAG TAC ATG ACC ACT TGA<br>K_d= 30 nM

Surface immobilization of PC (Photocleavable) linker and aptamer. The structure of the PC linker and the photolysis products generated following irradiation are shown in **Figure S2** (5-((7-((2-(2-(2-aminoethoxy)ethoxy)ethyl)(ethyl)amino)-2-oxo-2*H*-chromen-4-yl)methoxy)-5-oxo-pentanoic acid). Detailed synthetic routes and characterization of the PC linker used in this study are described elsewhere *(28)*. We verified the purity and cleavage of the PC linker used in this study with the results reported here. The PC linker was dissolved in PBS (~2.0 μM) and exposed to blue light (400-450 nm, 34 ±4 mW/cm^2). Samples were collected after 0, 2, and 10 min of light irradiation and the products produced by irradiation were analyzed by UPLC/HRMS (Waters Acquity UPLC with a photodiode array UV detector and an LCT Premiere TOF mass spectrometer). Two μL of the irradiated PC linker was injected into a Waters Acquity Atlantis T3 column (2.1 x 50 mm, 1.7 μm particle size) and the separation performed at a flow rate of 0.6 mL/min. The gradient was run over 3.0 min and consisted of water and acetonitrile (95:5 to 0:100 with 0.05% TFA). The detection wavelength was set at 247 nm.

Photolysis products (**Figure S2A**) were separated using UPLC (**Figure S2B**) and identified by mass spectrometry. The intact PC linker ((**1**), 81.2% arbitrary signal intensity, 1.2 min retention time) concentration decreased with irradiation time and at 2 min, the chromatographic peak for the intact PC linker disappeared. Major photolysis product (**2**) was detected at an arbitrary signal intensity of 4.2% (1 min retention time). Upon continued irradiation, this peak increased to 68% (arbitrary units). Not included in the calculation was product (**3**) because of its instability *(28)*. Before PC linker attachment, VP selection chips were UV/O_3 activated. EDC (20 mg/mL) and NHS (2 mg/mL) were dissolved in dry ACN and infused into the device using all-plastic Norm-Ject™ syringes (Air-Tite). Next, devices were wrapped in a protective Rubylith® film (Ulano) to prevent subsequent exposure of the PC linker to ambient light. After 25 min incubation at room temperature, reagents were displaced by air and the 1 mM PC linker (dissolved in dry ACN with a 2x excess of Triethylamine (TEA; 2 mM) was infused into the device via a vacuum pump.

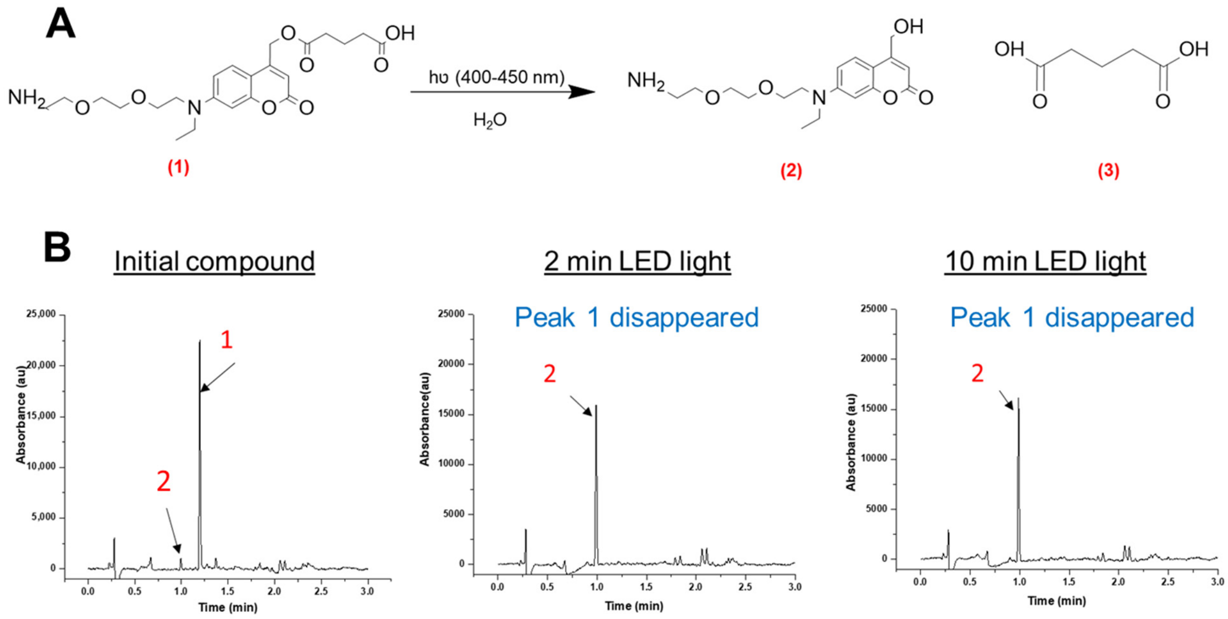

Figure S2. PC linker photolysis and products. (**A**) Photocleavage of 7-amino coumarin occurs from its meta-carbon after excitation, which forms an ion pair (coumarinylmethyl cation and a leaving conjugate base) as key intermediates. Coumarinylmethyl cation reacts with a nucleophile resulting in bond cleavage and release of the selected biomarker *(61)*. (**B**) Ultra-high performance liquid chromatography (UPLC) of the photocleavage of (**1**) using 400 – 450 nm light for exposure times of 0, 2, and 10 min.

After incubating for 2 h at room temperature, reagents were displaced by air and the device was infused with 100 mM Tris (pH 7.4) and incubated for 30 min at room temperature to inactivate any unreacted NHS ester groups on the device's surface. A second EDC/NHS reaction in ACN was performed to activate -COOH groups at the end of the PC linker. Following surface activation, an aptamer (40 μM)

dissolved in PBS (pH 7.4) was infused into a chip and incubated overnight at 4°C. When changing between anhydrous solvents and any buffered solution, devices were flushed with nuclease free water.

LED light exposure system. An 885 mW LED (M420L3, ThorLabs) producing light from 385–470 nm (λ_{max} = 412 nm) was filtered through a 400 nm longpass colored glass filter (Edmund Optics) that was used to photocleave the PC linker. The LED's innate divergence (60°) illuminated a 90 mm diameter spot at a 24 mm distance, which allowed irradiation of the entire VP selection chip. The power distribution was measured with an 18 mm x 18 mm power sensor (ThorLabs) rastered beneath the LED spot. For photocleavage reactions, the LED was mounted to a polished aluminum chamber with recesses that centered devices 24 mm beneath the LED and the LED was powered using an analog LED driver (Thorlabs).

Optical transmittance of thermoplastic used for the VP selection chip. The optical transmittance of COP (cyclic olefin polymer) and COC (cyclic olefin copolymer) polymers were evaluated using UV-vis spectroscopy to ensure that sufficient amounts of blue light penetrated the microfluidic cover plate and substrate to provide sufficient levels of photolysis of the PC linker to release the VPs efficiently. **Figure 4E** in the main manuscript shows the transmittance spectra of COP and COC polymers. COC and COP transmittance was ~85-90% at the peak LED emission wavelength. The spectral distribution of the LED used in these experiments was 385 – 470 nm (λ_{max} = 412 nm) and overlapped with the PC linker's absorption minimum (~400 nm). The LED was placed 24 mm from the selection chip's surface to allow the innate divergence of the beam to provide a spot diameter of 90 mm, which provided homogenous illumination (34 ±4 mW/cm^2) over the entire VP selection chip. While the 1% PVP/0.5% BSA solution used to block the microfluidic chip surface to prevent non-specific adsorption showed some absorption over the spectral distribution of the blue-LED (**Figure 4F** in the main manuscript), it was not used for the wash and release phases of VP assay and thus, the amount of absorption anticipated for a monolayer of PVP/BSA on the chip's surface would be negligible compared to the conditions used to acquire the spectrum shown in **Figure 4F**.

Blocking Buffers. Table S2 presents a summary for the evaluation of the efficiency of blocking agents in preventing non-specific adsorption of VPs to the selection chip's surfaces. A 1% polyvinylpyrrolidone (PVP) and 0.5% bovine serum albumin (BSA) in PBS as a blocking buffer showed the lowest levels of non-specific adsorption (1.4 ±0.1%).

Table S2. Efficacy of blocking buffers for preventing non-specific adsorption of VPs to the polymer surface.

	UV/O_3 activated device passivated with blocking agents			
	Bare surface (n=3)	1% BSA in PBS (n=3)	1% PVP/0.5% BSA in PBS (n=4)	2% PEG in PBS (n=4)
Viral Particles non-specific adsorption (%)	17.3 ±10.9	5.3 ±0.4	1.4 ±0.1	1.4 ±1.6

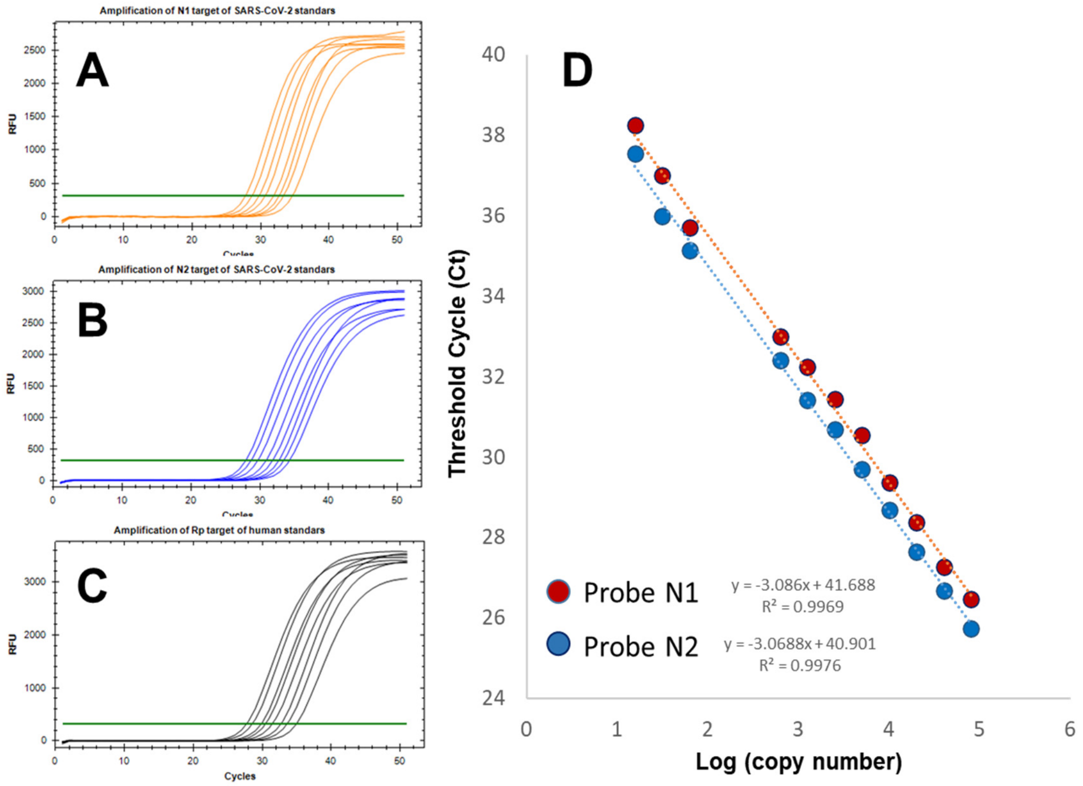

Figure S3. RT-qPCR amplification curves. The following genes are shown: (**A**) N1 gene; (**B**) N2 gene of SARS-CoV-2 viruses, and (**C**) human RppH gene. (**D**) Standard calibration curves for N1 and N2 genes of SARS-CoV-2.

Quantitative reverse transcription PCR (RT-qPCR). RT-qPCR was used as the standard method for assay optimization and validation and to secure quantitative results. For this, samples were subjected to RNA extraction using a Zymo viral RNA extraction kit following the manufacturer's protocol. Purified total RNA was eluted in ~10 μL of nuclease free water and profiles of extracted total RNA were analyzed and quantified using gel electrophoresis (Agilent 2200 HSRNA TapeStation). Following purification, total RNA was immediately used for cDNA synthesis (5 μL for each RT(+) and RT(-) reactions in 50 μL total volume).

cDNA was synthesized via reverse transcription (RT) with random primers using ProtoScript II First Strand cDNA Synthesis Kit according to the manufacturer's instructions. RT(-) control reactions were performed in the absence of the RT enzyme. The thermal program consisted of 25°C (5 min), 42°C for 1 h followed by an enzyme inactivation step of 80°C for 5 min. qPCR consisted of 4 μL of cDNA in a 20 μL reaction volume with 0.5 μM forward and reverse primers and 0.125 μM probe. The PCR thermal cycles for SARS-CoV-2 consisted of the CDC recommended protocol, which consisted of an initial denaturation step at 95°C for 3 min followed by 50 cycles of the following: 95°C for 3 s, 55°C for 30 s, and 72°C for 40 s. Typical amplification profiles for the N1 and N2 genes of SARS-CoV-2 and the human RppH gene are shown in **Figures S3A-C**, respectively.

RT-qPCR for HCoV OC43 and HCoV 229E were carried out using the following conditions: PCR activation at 95°C for 3 min and 50 cycles of amplification (15 s at 95°C and 1 min at 60°C). Calibration curves for N1 and N2 for SARS-CoV-2 samples are presented in **Figure S3D.** A summary of the qPCR figures-of-merit are shown in **Table S4.** Using HCoV 229E RT-PCR, viral RNA copies ranging from 200 to 2 × 10^9 per reaction were detected corresponding to 10^4 to 10^{11} viral genome equivalents per mL.

Table S3. Primers and probes sequences used in this study for the RT-qPCR.

Virus Name, Gene Name		Reversed Primer 5'- 3'	Forward Primer 5'- 3'	Probe FAM-5'- 3'-BHQ1
HRSV, F gene		CTTTTGATCTTGTTCACTTCTCCTTCT	TTGGATCTGCAATCGCCA	TGGCACTGCTGTATCTAAGGTCCTGCACT
SARS-CoV-2	N1	TCTGGTTACTGCCAGTTGAATCTG	GACCCCAAAATCAGCGAAAT	ACCCCGCATTACGTTTGGTGGACC
	N2	GCGCGACATTCCGAAGAA	TTACAAACATTGGCCGCAAA	ACAATTTGCCCCCAGCGCTTCAG
	RNAse	GAGCGGCTGTCTCCACAAGT	AGATTTGGACCTGCGAGCG	TTCTGACCTGAAGGCTCTGCGCG
HCoV OC43		AATGTAAAGATGGCCGCGTATT	ATGTTAGGCCGATAATTGAGGACTAT	CATACTCTGACGGTCACAAT
HCoV 229E		CCAACACGGTTGTGACAGTGA	TTCCGACGTGCTCGAACTTT	TCCTGAGGTCAATGCA

Table S4. **RT-qPCR figures-of-merit for the detection and quantification of different VPs.**

Virus Name	Dynamic range tested (gRNA copies)	Linear regression
**SARS-CoV-2**	N1 and N2: 12 x10^0 – 0.8x10^5	N1: y = -3.086x + 41.688 (R^2 = 0.997)<br>N2: y = -3.069x + 40.901 (R^2 = 0.997)
**HRSV**	12 x10^0 – 1.1 x10^5	y = -3.788x + 41.048 (R^2 = 0.996)
**HCoV OC43**	16 x10^0 – 0.4x10^5	y = -3.6048x + 41.956 (R^2 = 0.998)
**HCoV 229E**	16 x10^0 – 0.4x10^5	y = -3.8881x + 42.865 (R^2 = 0.996)

Surface plasmon resonance (SPR). SPR was performed using a BIAcore T200 SPR instrument with a CMD200L chip (Xantec Bioanalytics, GmbH; Dusseldorf, Germany). Data were collected at 25°C using a flow rate of 20 µL/min. The running buffer used in all experiments consisted of 10 mM HEPES/140 mM NaCl/0.005% Tween. Ten mM NaOH was used for regeneration of the surface.

Genomic RNA contamination in the VP selection chip. Viral particles present in the eluted fraction following PC linker cleavage was verified via AFM (**Figure S4A**). We also assessed free gRNA (RNA not encased within the viral envelop) non-specifically adsorbed onto the chip. gRNA isolated from SARS-CoV-2 VPs was passed at 0.8 mm/s (20 µL/min) through the VP selection chip containing the anti-S protein's 51-nt aptamer. Chips were washed, subjected to 2 min blue light exposure to cleave the PC linker, and the eluent was tested via RT-qPCR. Less than <0.3% gRNA was detected (**Figure S4B**).

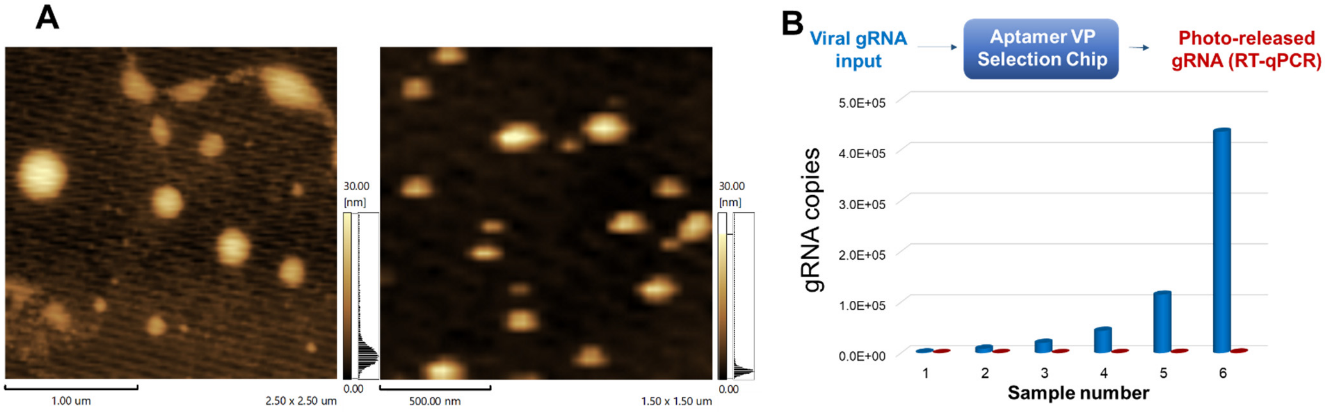

Figure S4. AFM images of SARS-CoV-2 and RNA adsorption to the VP selection chip. (**A**) AFM images of heat-inactivated SARS-CoV-2 particles that were selected using the VP selection chip and subsequently photo-released using a blue LED. The size of the released particles is similar to the size of the SARS-CoV-2 particles determined by NTA (see **Figure 3**). (**B**) gRNA was extracted from heat-inactivated SARS-CoV-2 particles, suspended in 1× PBS buffer and passed through the VP selection chip at 20 µL/min (copies between 1.3×10^3 and 4.2×10^5 were introduced in the VP selection chip). Bar graph showing the copies of gRNA found in the starting material (input, blue bars) and that found in the eluent (red bars) following release (0- 0.6×10^3 gRNA copies found). The gRNA copy numbers were quantified using RT-PCR.

Nanoparticle tracking analysis (NTA). VPs were analyzed via NTA (Nanosight NT 2.3). The samples were diluted 20× and vortexed prior to analysis. The instrument parameters used for the analysis consisted of: (i) Camera shutter 1206; (ii) camera gain 366; and (iii) capture duration 60 s. Five videos were taken for each sample at 25°C. Typical NTA traces for heat inactivated SARS-CoV-2 VPs from a stock solution and VPs that were selected using the VP selection chip and subsequently released are shown in **Figure 3D**. The flow cell of the Nanosight instrument was washed 5 times with PBS in between sample analyses. During the final wash with PBS, a video was monitored to check if there were any

particles left in the flow cell. If particles were detected, washing was continued until no particles were seen.

Scanning Electron Microscope. SEMs of the thermoplastic microfluidic devices were acquired using a Hitachi FlexSEM 1000 II SEM. The thermoplastics were sputter coated with a 10 nm conductive Au layer prior to SEM using a Denton Desk II Sputter Coater.

Transmission Electron Microscope (TEM) images of selected and released VPs. The negative stain TEMs were conducted at the Scripps Core Microscopy Facility. Carbon-coated copper grids (400 mesh) were glow-discharged, and 10 µl of each sample was adsorbed to the grid for 2 min. Excess sample was wicked away, and grids were negatively stained with 2% uranyl formate for 2 min. Excess stain was wicked away, and the grids were allowed to dry. Samples were analyzed at 120 kV with a Talos L120C TEM (Thermo Fisher Scientific) and images were acquired with a CETA 16M complementary metal-oxide semiconductor (CMOS) camera. See **Figure S5** for images.

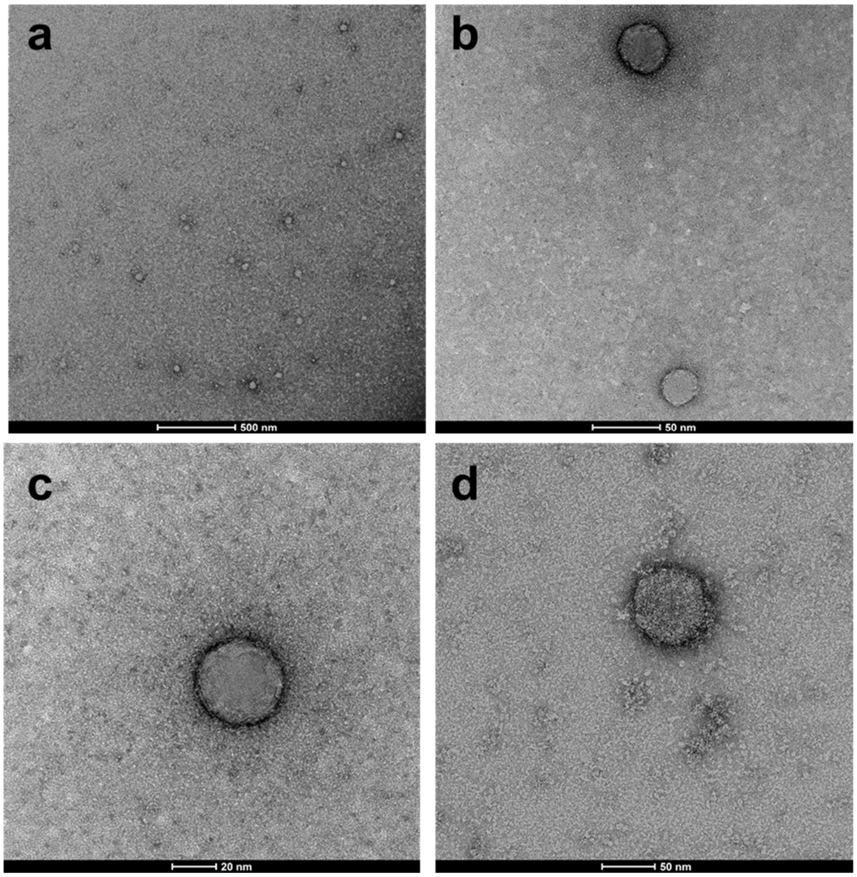

Figure S5. TEM images of affinity selected heat-inactivated SARS-CoV-2 virions from saliva that were subsequently photo-released from the VP selection chip. Images were collected using a magnification of: (a) 22,000x, (b) 92,000x, and (c, d) 150,000x following negative staining with 2% uranyl formate.

Viral Genomic RNA gel electrophoresis. gRNA isolated from VPs was purified using a spin column and eluted from the column in ~8 μL of water. Two μL of the eluent was taken for RNA quantification, using the Tape-station gel electrophoresis system. The gel images are shown in **Figure S6**. The majority of the gRNA was the full length 30 knt RNA and minute amounts of two small fragments were seen.

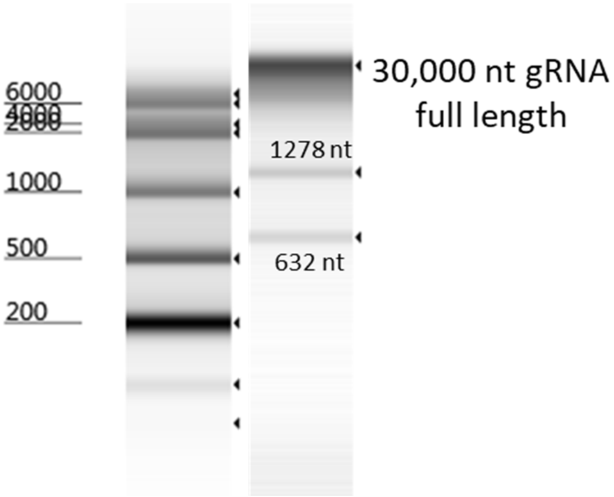

Figure S6. Gel images of gRNA extracted from affinity released heat-inactivated SARS-CoV-2 particles. The gRNA was acquired from affinity-selected VPs using the VP selection chip and subsequently photo-released using a blue LED.

Saliva samples. About 2 mL of saliva was collected from consenting adults according to an approved IRB protocol and placed into sterile plastic tubes containing no stabilizer. The saliva was then aliquoted into 200 μL volumes and stored at -80°C until further analysis was required. All patient samples were de-identified before sending to the laboratory for analysis.

Clinical sample testing. The process flow for testing 30 clinical samples is shown in **Figure 5B**. Details about patient's diagnosis time and testing times are given in **Table S5.** Ten de-identified saliva samples from volunteers that were asymptomatic were subsequently tested to assure they were COVID-19 negative (these samples were confirmed to be COVID-19 negative by RT-qPCR). Saliva samples were centrifuged at 1,000 x g for 5 min. before processing on the VP selection chip.

Ten SARS-CoV-2 gRNA positive saliva samples were received from individuals admitted to the University of Kansas Hospital between late July and early September 2020 for severe COVID-19-related symptoms. Upon admission, a nasopharyngeal sample was collected, and the samples were subjected to RT-qPCR to confirm COVID-19 positive status. For these patients, the level of neutralizing antibodies was measured as well. The de-identified samples were blinded and shipped to the testing laboratory on the KU-Lawrence campus. Another set of 10 saliva samples were collected from healthy volunteers at KUMC (October 2020). An additional five samples in December 2020 and another 5 in July 2021 were

collected from symptomatic patients undergoing SARS-CoV-2 testing through the Wyandotte Health Department (sample ID #21-25) and the KU Hospital (#26-30), respectively. None required hospital stays. For these patients, the level of neutralizing antibodies was not measured. All saliva samples were stored at -80°C until required for measurements.

Table S5. Clinical samples evaluated in the study.

Sample ID	Patient ID	Date of 1st COVID-19 symptoms	Saliva Sample Collection Date	COVID-19 Test*
1	1	-	10/26/2020	negative
4	4	-	10/26/2020	negative
5	5	-	10/26/2020	negative
7	7	-	10/27/2020	negative
8	8	-	10/27/2020	negative
10	10	-	10/27/2020	negative
13	13	-	10/28/2020	negative
14	14	-	10/28/2020	negative
16	16	-	10/28/2020	negative
19	19	-	10/28/2020	negative
2	101	07/25/2020	8/7/2020	positive
3	201	07/21/2020	8/3/2020	positive
6	102	08/02/2020	8/11/2020	positive
9	103	08/11/2020	8/17/2020	positive
11	203	09/02/2020	9/12/2020	positive
12	104	08/17/2020	8/21/2020	positive
15	204	09/02/2020	9/14/2020	positive
17	105	09/02/2020	9/16/2020	positive
18	205	09/07/2020	9/19/2020	positive
20	106	09/09/2020	9/20/2020	positive
21	036697	na	12/2020	positive
22	036699	na	12/2020	positive
23	036700	na	12/2020	positive
24	036721	na	12/2020	positive
25	036722	na	12/2020	positive
26	603190	na	08/2021	positive
27	603280	na	08/2021	positive
28	603345	na	08/2021	positive
29	603355	na	08/2021	positive
30	603356	na	08/2021	positive

Note: *- COVID-19 positive individuals' status was confirmed using the Cepheid Xpress SARS-CoV-2 test from Nasopharyngeal swabs.

REFERENCES AND NOTES

1. Z. Ke, J. Oton, K. Qu, M. Cortese, V. Zila, L. McKeane, T. Nakane, J. Zivanov, C. J. Neufeldt, B. Cerikan, J. M. Lu, J. Peukes, X. Xiong, H.-G. Kräusslich, S. H. W. Scheres, R. Bartenschlager, J. A. G. Briggs, Structures and distributions of SARS-CoV-2 spike proteins on intact virions. *Nature* **588**, 498–502 (2020).

2. M. J. Mina, R. Parker, D. B. Larremore, Perspective: Rethinking Covid-19 test sensitivity—A strategy for containment. *N. Engl. J. Med.* **383**, e120 (2020).

3. CDC, COVID-19. (2021), vol. 2021; https://www.cdc.gov/coronavirus/2019-ncov/index.html.

4. C. A. Hogan, N. Garamani, A. S. Lee, J. K. Tung, M. K. Sahoo, C. Huang, B. Stevens, J. Zehnder, B. A. Pinsky, Comparison of the Accula SARS-CoV-2 test with a laboratory-developed assay for detection of SARS-CoV-2 RNA in clinical nasopharyngeal specimens. *J. Clin. Microbiol.* **58** e01072-20 (2020).

5. H. M. Creager, B. Cabrera, A. Schnaubelt, J. L. Cox, A. M. Cushman-Vokoun, S. M. Shakir, K. D. Tardif, M.-L. Huang, K. R. Jerome, A. L. Greninger, D. Drobysheva, U. Spaulding, M. Rogatcheva, K. M. Bourzac, S. H. Hinrichs, M. J. Broadhurst, P. D. Fey, Clinical evaluation of the BioFire® Respiratory Panel 2.1 and detection of SARS-CoV-2. *J. Clin. Virol.* **129**, 104538 (2020).

6. B. Visseaux, Q. Le Hingrat, G. Collin, D. Bouzid, S. Lebourgeois, D. Le Pluart, L. Deconinck, F.-X. Lescure, J.-C. Lucet, L. Bouadma, J.-F. Timsit, D. Descamps, Y. Yazdanpanah, E. Casalino, N. Houhou-Fidouh; Emergency Department Influenza Study Group, Evaluation of the QIAstat-Dx Respiratory SARS-CoV-2 Panel, the first rapid multiplex PCR commercial assay for SARS-CoV-2 detection. *J. Clin. Microbiol.* **58**, e00630-20 (2020).

7. D. Nörz, N. Fischer, A. Schultze, S. Kluge, U. Mayer-Runge, M. Aepfelbacher, S. Pfefferle, M. Lütgehetmann, Clinical evaluation of a SARS-CoV-2 RT-PCR assay on a fully automated system for rapid on-demand testing in the hospital setting. *J. Clin. Virol.* **128**, 104390 (2020).

8. A. Hosseini, R. Pandey, E. Osman, A. Victorious, F. Li, T. Didar, L. Soleymani, Roadmap to the bioanalytical testing of COVID-19: From sample collection to disease surveillance. *ACS Sens.* **5**, 3328–3345 (2020).

9. Q. Matthews, S. J. R. da Silva, M. Norouzi, L. J. Pena, K. Pardee, Adaptive, diverse and de-centralized diagnostics are key to the future of outbreak response. *BMC Biol.* **18**, 153 (2020).

10. P. Mertens, N. De Vos, D. Martiny, C. Jassoy, A. Mirazimi, L. Cuypers, S. Van den Wijngaert, V. Monteil, P. Melin, K. Stoffels, N. Yin, D. Mileto, S. Delaunoy, H. Magein, K. Lagrou, J. Bouzet, G. Serrano, M. Wautier, T. Leclipteux, M. Van Ranst, O. Vandenberg; LHUB-ULB SARS-CoV-2 Working Diagnostic Group, Development and potential usefulness of the COVID-19 Ag Respi-Strip diagnostic assay in a pandemic context. *Front. Med.* **7**, 225 (2020).

11. X. He, E. H. Y. Lau, P. Wu, X. Deng, J. Wang, X. Hao, Y. C. Lau, J. Y. Wong, Y. Guan, X. Tan, X. Mo, Y. Chen, B. Liao, W. Chen, F. Hu, Q. Zhang, M. Zhong, Y. Wu, L. Zhao, F. Zhang, B. J. Cowling, F. Li, G. M. Leung, Temporal dynamics in viral shedding and transmissibility of COVID-19. *Nat. Med.* **26**, 672–675 (2020).

12. J. A. Berkenbrock, R. Grecco-Machado, S. Achenbach, Microfluidic devices for the detection of viruses: Aspects of emergency fabrication during the COVID-19 pandemic and other outbreaks. *Proc. Math. Phys. Eng. Sci.* **476**, 20200398 (2020).

13. A. Basiri, A. Heidari, M. F. Nadi, M. T. P. Fallahy, S. S. Nezamabadi, M. Sedighi, A. Saghazadeh, N. Rezaei, Microfluidic devices for detection of RNA viruses. *Rev. Med. Virol.* **31**, 1–11 (2021).

14. C. D. M. Campos, K. Childers, S. S. T. Gamage, H. Wijerathne, Z. Zhao, S. A. Soper, Analytical technologies for liquid biopsy of subcellular materials. *Annu. Rev. Anal. Chem.* **14**, 207–229 (2021).

15. G. Seo, G. Lee, M. J. Kim, S.-H. Baek, M. Choi, K. B. Ku, C.-S. Lee, S. Jun, D. Park, H. G. Kim, S.-J. Kim, J.-O. Lee, B. T. Kim, E. C. Park, S. I. Kim, Rapid detection of COVID-19

causative virus (SARS-CoV-2) in human nasopharyngeal swab specimens using field-effect transistor-based biosensor. *ACS Nano* **14**, 5135–5142 (2020).

16. M. Alafeef, P. Moitra, K. Dighe, D. Pan, RNA-extraction-free nano-amplified colorimetric test for point-of-care clinical diagnosis of COVID-19. *Nat. Protoc.* **16**, 3141–3162 (2021).

17. P. Moitra, M. Alafeef, K. Dighe, M. B. Frieman, D. Pan, Selective naked-eye detection of SARS-CoV-2 mediated by n gene targeted antisense oligonucleotide capped plasmonic nanoparticles. *ACS Nano* **14**, 7617–7627 (2020).

18. D. Wrapp, N. Wang, K. S. Corbett, J. A. Goldsmith, C.-L. Hsieh, O. Abiona, B. S. Graham, J. S. McLellan, Cryo-EM structure of the 2019-nCoV spike in the prefusion conformation. *Science* **367**, 1260–1263 (2020).

19. E. Callaway, Heavily mutated Omicron variant puts scientists on alert. *Nature* **600**, 21 (2021).

20. A. D. Ellington, J. W. Szostak, In vitro selection of RNA molecules that bind specific ligands. *Nature* **346**, 818–822. (1990).

21. C. Tuerk, L. Gold, Systematic evolution of ligands by exponential enrichment: RNA ligands to bacteriophage T4 DNA polymerase. *Science* **249**, 505–510. (1990).

22. S. M. Nimjee, C. P. Rusconi, B. A. Sullenger, Aptamers: An emerging class of therapeutics. *Annu. Rev. Med.* **56**, 555–583. (2005).

23. L. Gold, B. Polisky, O. Uhlenbeck, M. Yarus, Diversity of oligonucleotide functions. *Annu. Rev. Biochem.* **64**, 763–797. (1995).

24. A. S. Peinetti, R. J. Lake, W. Cong, L. Cooper, Y. Wu, Y. Ma, G. T. Pawel, M. E. Toimil-Molares, C. Trautmann, L. Rong, B. Mariñas, O. Azzaroni, Y. Lu, Direct detection of human adenovirus or SARS-CoV-2 with ability to inform infectivity using DNA aptamer-nanopore sensors. *Sci. Adv.* **7**, eabh2848 (2021).

25. A. Schmitz, A. Weber, M. Bayin, S. Breuers, V. Fieberg, M. Famulok, G. Mayer, A SARS-CoV-2 spike binding DNA aptamer that inhibits pseudovirus infection by an RBD-independent mechanism**. *Angew. Chem. Int. Ed.* **60**, 10279–10285 (2021).

26. Y. Song, J. Song, X. Wei, M. Huang, M. Sun, L. Zhu, B. Lin, H. Shen, Z. Zhu, C. Yang, Discovery of aptamers targeting the receptor-binding domain of the SARS-CoV-2 spike glycoprotein. *Anal. Chem.* **92**, 9895–9900 (2020).

27. M. Sun, S. Liu, X. Wei, S. Wan, M. Huang, T. Song, Y. Lu, X. Weng, Z. Lin, H. Chen, Y. Song, C. Yang, Aptamer blocking strategy inhibits SARS-CoV-2 virus infection. *Angew. Chem. Int. Ed. Engl.* **60**, 10266–10272 (2021).

28. T. N. Pahattuge, J. M. Jackson, R. Digamber, H. Wijerathne, V. Brown, M. A. Witek, C. Perera, R. S. Givens, B. R. Peterson, S. A. Soper, Visible photorelease of liquid biopsy markers following microfluidic affinity-enrichment. *Chem. Commun.* **56**, 4098–4101 (2020).

29. H. Becker, It’s the economy. *Lab Chip* **9**, 2759–2762 (2009).

30. H. Wijerathne, M. A. Witek, J. M. Jackson, V. Brown, M. L. Hupert, K. Herrera, C. Kramer, A. E. Davidow, Y. Li, A. E. Baird, M. C. Murphy, S. A. Soper, Affinity enrichment of extracellular vesicles from plasma reveals mRNA changes associated with acute ischemic stroke. *Commun. Biol.* **3**, 613 (2020).

31. A. K. Trilling, J. Beekwilder, H. Zuilhof, Antibody orientation on biosensor surfaces: A minireview. *Analyst* **138**, 1619 (2013).

32. S. Yang, H. Li, L. Xu, Z. Deng, W. Han, Y. Liu, W. Jiang, Y. Zu, Oligonucleotide aptamer-mediated precision therapy of hematological malignancies. *Mol. Ther. Nucleic Acids* **13**, 164–175 (2018).

33. Z. Chai, L. Guo, H. Jin, Y. Li, S. Du, Y. Shi, C. Wang, W. Shi, J. He, TBA loop mapping with 3′-inverted-deoxythymidine for fine-tuning of the binding affinity for α-thrombin. *Org. Biomol. Chem.* **17**, 2403–2412 (2019).

34. S. Y. Ni, H. Yao, L. Wang, J. Lu, F. Jiang, A. Lu, G. Zhang, Chemical modifications of nucleic acid aptamers for therapeutic purposes. *Int. J. Mol. Sci.* **18**, 1683 (2017).

35. Y. Pan, D. Zhang, P. Yang, L. L. M. Poon, Q. Wang, Viral load of SARS-CoV-2 in clinical samples. *Lancet Infect. Dis.* **20**, 411–412 (2020).

36. M. A. Witek, R. D. Aufforth, H. Wang, J. W. Kamande, J. M. Jackson, S. R. Pullagurla, M. L. Hupert, J. Usary, W. Z. Wysham, D. Hilliard, S. Montgomery, V. Bae-Jump, L. A. Carey, P. A. Gehrig, M. I. Milowsky, C. M. Perou, J. T. Soper, Y. E. Whang, J. J. Yeh, G. Martin, S. A. Soper, Discrete microfluidics for the isolation of circulating tumor cell subpopulations targeting fibroblast activation protein alpha and epithelial cell adhesion molecule. *NPJ Precis. Oncol.* **1**, 24 (2017).

37. A. A. Pater, M. S. Bosmeny, C. L. Barkau, K. N. Ovington, R. Chilamkurthy, M. Parasrampuria, S. B. Eddington, A. O. Yinusa, A. A. White, P. E. Metz, R. J. Sylvain, M. M. Hebert, S. W. Benzinger, K. Sinha, K. T. Gagnon, Emergence and evolution of a prevalent new SARS-CoV-2 variant in the United States. bioRxiv 2021.01.11.426287 [**Preprint**]. 13 January 2021. https://doi.org/10.1101/2021.01.11.426287.

38. Y. Shu, J. McCauley, GISAID: Global initiative on sharing all influenza data - From vision to reality. *Euro Surveill.* **22**, (2017).

39. M. Scudellari, How the coronavirus infects cells - And why Delta is so dangerous. *Nature* **595**, 640–644 (2021).

40. A. Tong, T. C. Sorrell, A. J. Black, C. Caillaud, W. Chrzanowski, E. Li, D. Martinez-Martin, A. McEwan, R. Wang, A. Motion, A. C. Bedoya, J. Huang, L. Azizi, B. J. Eggleton; COVID-19 Sensor Research Priority-Setting Investigators, Research priorities for COVID-19 sensor technology. *Nat. Biotechnol.* **39**, 144–147 (2021).

41. C. B. Jackson, M. Farzan, B. Chen, H. Choe, Mechanisms of SARS-CoV-2 entry into cells. *Nat. Rev. Mol. Cell Biol.* **23**, 3–20 (2021).

42. S. K. Brooks, R. K. Webster, L. E. Smith, L. Woodland, S. Wessely, N. Greenberg, G. J. Rubin, The psychological impact of quarantine and how to reduce it: Rapid review of the evidence. *Lancet* **395**, 912–920 (2020).

43. C. Maringe, J. Spicer, M. Morris, A. Purushotham, E. Nolte, R. Sullivan, B. Rachet, A. Aggarwal, The impact of the COVID-19 pandemic on cancer deaths due to delays in diagnosis in England, UK: A national, population-based, modelling study. *Lancet* **21**, 1023–1034 (2020).

44. K. K.-W. To, O. T.-Y. Tsang, C. C.-Y. Yip, K.-H. Chan, T.-C. Wu, J. M.-C. Chan, W.-S. Leung, T. S.-H. Chik, C. Y.-C. Choi, D. H. Kandamby, D. C. Lung, A. R. Tam, R. W.-S. Poon, A. Y.-F. Fung, I. F.-N. Hung, V. C.-C. Cheng, J. F.-W. Chan, K.-Y. Yuen, Consistent detection of 2019 novel coronavirus in saliva. *Clin. Infect. Dis.* **71**, 841–843 (2020).

45. M. H. Katz, Challenges in testing for SARS-CoV-2 among patients who recovered from COVID-19. *JAMA Intern. Med.* **181**, 704–705 (2021).

46. H. Kang, Y. Wang, Z. Tong, X. Liu, Retest positive for SARS-CoV-2 RNA of "recovered" patients with COVID-19: Persistence, sampling issues, or re-infection? *J. Med. Virol.* **92**, 2263–2265 (2020).

47. N. Li, X. Wang, T. Lv, Prolonged SARS-CoV-2 RNA shedding: Not a rare phenomenon. *J. Med. Virol.* **92**, 2286–2287 (2020).

48. J. Bullard, K. Dust, D. Funk, J. E. Strong, D. Alexander, L. Garnett, C. Boodman, A. Bello, A. Hedley, Z. Schiffman, K. Doan, N. Bastien, Y. Li, P. G. V. Caeseele, G. Poliquin, Predicting infectious severe acute respiratory syndrome coronavirus 2 from diagnostic samples. *Clin. Infect. Dis.* **71**, 2663–2666 (2020).

49. M. Nagura-Ikeda, K. Imai, S. Tabata, K. Miyoshi, N. Murahara, T. Mizuno, M. Horiuchi, K. Kato, Y. Imoto, M. Iwata, S. Mimura, T. Ito, K. Tamura, Y. Kato, Clinical evaluation of self-collected saliva by RT-qPCR, direct RT-qPCR, RT-LAMP, and a rapid antigen test to diagnose COVID-19. *J. Clin. Microbiol.* **58**, e01438-20 (2020).

50. P. Kalra, A. Dhiman, W. C. Cho, J. G. Bruno, T. K. Sharma, Simple methods and rational design for enhancing aptamer sensitivity and specificity. *Front. Mol. Biosci.* **5**, 41–41 (2018).

51. W. F. Garcia-Beltran, K. J. St. Denis, A. Hoelzemer, E. C. Lam, A. D. Nitido, M. L. Sheehan, C. Berrios, O. Ofoman, C. C. Chang, B. M. Hauser, J. Feldman, A. L. Roederer, D. J. Gregory, M. C. Poznansky, A. G. Schmidt, A. J. Iafrate, V. Naranbhai, A. B. Balazs, mRNA-based COVID-19 vaccine boosters induce neutralizing immunity against SARS-CoV-2 Omicron variant. *Cell* **185**, 457–466.e4 (2022).

52. D. S. Khoury, D. Cromer, A. Reynaldi, T. E. Schlub, A. K. Wheatley, J. A. Juno, K. Subbarao, S. J. Kent, J. A. Triccas, M. P. Davenport, Neutralizing antibody levels are highly predictive of immune protection from symptomatic SARS-CoV-2 infection. *Nat. Med.* **27**, 1205–1211 (2021).

53. S. Dispinseri, M. Secchi, M. F. Pirillo, M. Tolazzi, M. Borghi, C. Brigatti, M. L. De Angelis, M. Baratella, E. Bazzigaluppi, G. Venturi, F. Sironi, A. Canitano, I. Marzinotto, C. Tresoldi, F. Ciceri, L. Piemonti, D. Negri, A. Cara, V. Lampasona, G. Scarlatti, Neutralizing antibody responses to SARS-CoV-2 in symptomatic COVID-19 is persistent and critical for survival. *Nat. Commun.* **12**, 2670 (2021).

54. E. Pasomsub, S. P. Watcharananan, K. Boonyawat, P. Janchompoo, G. Wongtabtim, W. Suksuwan, S. Sungkanuparph, A. Phuphuakrat, Saliva sample as a non-invasive specimen for the diagnosis of coronavirus disease 2019: A cross-sectional study. *Clin. Microbiol. Infect.* **27**, 285.e1–285.e4 (2020).

55. Y. G. Kim, S. G. Yun, M. Y. Kim, K. Park, C. H. Cho, S. Y. Yoon, M. H. Nam, C. K. Lee, Y. J. Cho, C. S. Lim, Comparison between saliva and nasopharyngeal swab specimens for detection of respiratory viruses by multiplex reverse transcription-PCR. *J. Clin. Microbiol.* **55**, 226–233 (2017).

56. M. L. Bastos, S. Perlman-Arrow, D. Menzies, J. R. Campbell, The sensitivity and costs of testing for SARS-CoV-2 infection with saliva versus nasopharyngeal swabs. *Ann. Intern. Med.* **174**, 501–510 (2021).

57. Z. D. Harms, K. B. Mogensen, P. S. Nunes, K. Zhou, B. W. Hildenbrand, I. Mitra, Z. Tan, A. Zlotnick, J. P. Kutter, S. C. Jacobson, Nanofluidic devices with two pores in series for resistive-pulse sensing of single virus capsids. *Anal. Chem.* **83**, 9573–9578 (2011).

58. L. Miao, J. Li, Q. Liu, R. Feng, M. Das, C. M. Lin, T. J. Goodwin, O. Dorosheva, R. Liu, L. Huang, Transient and local expression of chemokine and immune checkpoint traps to treat pancreatic cancer. *ACS Nano* **11**, 8690–8706 (2017).

59. K. Percze, Z. Szakács, É. Scholz, J. András, Z. Szeitner, C. H. van den Kieboom, G. Ferwerda, M. I. de Jonge, R. E. Gyurcsányi, T. Mészáros, Aptamers for respiratory syncytial virus detection. *Sci. Rep.* **7**, 42794 (2017).

60. S. A. Soper, S. M. Ford, S. Qi, R. L. McCarley, K. Kelly, M. C. Murphy, Peer Reviewed: Polymeric microelectromechanical systems. *Anal. Chem.* **72**, 642 A–651 A (2000).

61. P. Klan, T. Solomek, C. G. Bochet, A. Blanc, R. Givens, M. Rubina, V. Popik, A. Kostikov, J. Wirz, Photoremovable protecting groups in chemistry and biology: Reaction mechanisms and efficacy. *Chem. Rev.* **113**, 119–191 (2013).
